# Planning ahead: Predictable switching recruits task‐active and resting‐state networks

**DOI:** 10.1002/hbm.26430

**Published:** 2023-07-20

**Authors:** Danielle L. Kurtin, Garazi Araña‐Oiarbide, Romy Lorenz, Ines R. Violante, Adam Hampshire

**Affiliations:** ^1^ NeuroModulation Lab, Department of Psychology, Faculty of Health and Medical Sciences University of Surrey Guildford UK; ^2^ Department of Brain Sciences, Faculty of Medicine Imperial College London London UK; ^3^ MRC Cognition and Brain Sciences Unit University of Cambridge Cambridge UK; ^4^ The Poldrack Lab Stanford University Stanford California USA; ^5^ Department of Neurophysics Max‐Planck Institute for Human Cognitive and Brain Sciences Leipzig Germany

**Keywords:** default mode network, fMRI, functional connectivity, multiple demand network, mutual information, switching, temporal predictability

## Abstract

Switching is a difficult cognitive process characterised by costs in task performance; specifically, slowed responses and reduced accuracy. It is associated with the recruitment of a large coalition of task‐positive regions including those referred to as the multiple demand cortex (MDC). The neural correlates of switching not only include the MDC, but occasionally the default mode network (DMN), a characteristically task‐negative network. To unpick the role of the DMN during switching we collected fMRI data from 24 participants playing a switching paradigm that perturbed predictability (i.e., cognitive load) across three switch dimensions—sequential, perceptual, and spatial predictability. We computed the activity maps unique to switch vs. stay trials and all switch dimensions, then evaluated functional connectivity under these switch conditions by computing the pairwise mutual information functional connectivity (miFC) between regional timeseries. Switch trials exhibited an expected cost in reaction time while sequential predictability produced a significant benefit to task accuracy. Our results showed that switch trials recruited a broader activity map than stay trials, including regions of the DMN, the MDC, and task‐positive networks such as visual, somatomotor, dorsal, salience/ventral attention networks. More sequentially predictable trials recruited increased activity in the somatomotor and salience/ventral attention networks. Notably, changes in sequential and perceptual predictability, but not spatial predictability, had significant effects on miFC. Increases in perceptual predictability related to decreased miFC between control, visual, somatomotor, and DMN regions, whereas increases in sequential predictability *increased* miFC between regions in the same networks, as well as regions within ventral attention/ salience, dorsal attention, limbic, and temporal parietal networks. These results provide novel clues as to how DMN may contribute to executive task performance. Specifically, the improved task performance, unique activity, and increased miFC associated with increased sequential predictability suggest that the DMN may coordinate more strongly with the MDC to generate a temporal schema of upcoming task events, which may attenuate switching costs.

## Graphical abstract

Increasing sequential predictability switching contributes to improved task performance, increased brain activity, and elevates functional connectivity between multiple demand cortex (MDC) and default mode network (DMN) regions. DMN and MDC coordination may reflect a working memory strategy whereby a temporal schema of upcoming switches is generated to attenuate switch costs.

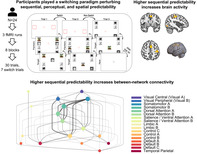

## 1 INTRODUCTION

Switching is an umbrella term that captures the various ways the performance of an ongoing task can be changed. For example, switches may be driven exogenously or organised endogenously, or a person may switch between stimulus and response rules, current goals within the same overarching task, or between different tasks altogether (Kim et al., 2011). The effects of switching on task performance are among the most established cognitive phenomena, being characterised by post‐switch costs in both reaction time (RT) and accuracy (Monsell, 2003). While the effects of switching on performance are well‐established, the exact set of brain regions influenced by switches may differ depending on the type of switch (Hampshire et al., 2016). However, it is almost invariably the case that meeting the demands of a switch is cognitively costly, and that coincident with the performance of a switch there is increased magnitude of BOLD response across large‐scale distributed networks of brain regions (Kimberg et al., 2000; Rushworth et al., 2002) and altered network functional connectivity (O'Connell & Basak, 2018; Qiao et al., 2020) as compared to sequences of trials where rules and requirements “stay” and are more uniform. These neural correlates of switching are often localised in the multiple demand cortex (MDC) (Camilleri et al., 2018; Stiers et al., 2010; Wang et al., 2021), which consists of the anterior insula, pre‐supplementary motor area (pre‐SMA), dorsolateral prefrontal cortex (dlPFC), inferior parietal sulcus (iPS), and dorsal anterior cingulate cortex, all of which are in salience, control, and frontoparietal networks. The MDC plays a broad role under conditions of heightened cognitive demand, such as planning what steps are needed to achieve task‐related goals (Duncan, 2010), rapidly reconfiguring to meet shifting task demands (Woolgar et al., 2011), and fine‐tuning its connectivity in response to increased cognitive load (Soreq et al., 2019). Thus, it is unsurprising that MDC is associated with switching performance.

Compared to MDC, the role of the default mode network (DMN) is less well understood. The DMN has historically been associated with processes that primarily occur during resting state, such as autobiographical recall and episodic memory (Fox et al., 2005; Spreng & Andrews‐Hanna, 2015). The DMN and MDC typically exhibit an anticorrelated relationship, where the DMN becomes more deactivated as the general cognitive difficulty of the task that a participant is engaged in increases (Mayer et al., 2010; McKiernan et al., 2003). Despite this deactivation during active task performance, disruptions in the MDC–DMN balance are nonetheless associated with cognitive deficits. For example, abnormal DMN coupling with MDC regions in neurological and psychiatric populations is associated with poorer cognitive task performance (Miskowiak & Petersen, 2019; Sambataro et al., 2010).

Consequently, it is notable that the DMN has also been reported to show increased activation under some conditions when switches are performed, for example, between different tasks (Crittenden et al., 2015; Lemire‐Rodger et al., 2019; Smith et al., 2018). The cases where regions in the DMN coactivate and show increased FC with task‐positive networks that they characteristically anticorrelate with (Buckner & DiNicola, 2019; Fox et al., 2005; Fransson, 2005) are often associated with modulations in task difficulty, offering a potential clue as to how these networks coordinate to support performance of complex tasks. For example, a study by O'Connell and Basak (2018) showed that connectivity between regions in the DMN and MDC increased with the difficulty of cognitive control paradigms that manipulated switching, maintenance, and updating. Conjoint MDC and DMN activation have also been observed during instruction‐based learning (IBL), when attention is initially oriented towards a new instruction (Hampshire et al., 2019) and the rule must be effortfully mapped into working memory.

While evidence has accumulated that the MDC and DMN may coordinate cognitive resources to meet the demands of tasks, it remains unclear what aspects of switching the DMN and MDC regions are most sensitive to. Here we developed a novel switching paradigm to further investigate how DMN–MDC network dynamics vary under conditions where three dimensions of switching predictability may be leveraged within the task schema in order to potentially mitigate the performance switch costs. The dimensions of switching predictability perturbed in this task include (1) sequential predictability, when within the sequence of event the switches occur, (2) spatial predictability, where the focus of attention will switch to next, and (3) perceptual predictability, what visual category the focus of attention will switch to next. We modulated the predictability of each dimension in a fully factorial design to evaluate whether predictability level influenced behavioural performance, network activity, and connectivity. By varying predictability, we manipulated cognitive load by enabling or disabling the construction of a mental schema of the task structure (Hunter 2020; Hunter 2021; Özbozdağlı et al., 2018). More sequentially, spatially, and perceptually predictable trials allow participants to construct a representation of when upcoming task switches will occur, where the new task will be located, and the visual characteristics of the next trial, respectively. This would enable participants to anticipate aspects of a task switch, prepare cognitive resources accordingly, and reduce switch costs (Hakun & Ravizza, 2012; Meiran, 1996; Meiran, 2000).

With this paradigm, we first confirm whether both DMN and MDC are more active during exogenously evoked switching. Then, we determine whether DMN–MDC networks dynamics are differentially sensitive to any specific aspect of switching predictability. Given the recency of work showing the DMN's engagement during switching we first sought to replicate this relationship, and if present, explore which/whether any of the switch dimensions influence the changes in the DMN–MDC relationship.

## 2 METHODS

### 2.1 Participants

We recruited 24 healthy volunteers (16 females, mean age 26.08 ± 4.0 years, standard deviation, two left‐handed). Participants reported no history of neurological or psychiatric conditions and had normal or corrected vision. The Hammersmith and Queen Charlotte's Research Ethics committee approved the studies, which conform with the Declaration of Helsinki. All participants gave their written consent after being informed about the nature of the study.

### 2.2 Switching paradigm

The switching task was programmed in MATLAB using Psychophysics Toolbox extension (Brainard, 1997), based on a previously reported simple IBL paradigm (Hampshire et al., 2016; Hampshire et al., 2019). The goal of the task was for participants to perform a binary‐discrimination exercise where they matched a target image to one of the previously presented flanker stimuli (rules). The task started with a rest period (16 s) indicated by the presence of a black fixation cross centred in the middle of a white screen. Next, two red rectangles were displayed on either side of the screen with a central red fixation cross for 500 ms, which indicated the participants to the location of the coming relevant stimuli. After this, four pairs of flanker images were presented for 4 s. The stimuli that composed the flanker images were created by Hampshire et al. (2008) and consisted of four categories of images: male faces, abstract lines, abstract figures, and rooms. The four pairs of flanker images contained one pair from each category. During presentation of the flanker images, the red fixation cross remained presented in the screen to direct the participants to the relevant set of flankers. After 4 s, the flankers of interest (i.e., the two images and the fixation cross) were replaced by a target image centred on the fixation cross. The target image was of 1° of similarity to one of the flanker stimuli (either the left or right in the row participants were instructed to attend to). The varying degrees of similarity between the flanker and target images were created by morphing within a category. The participants were asked to select the flanker that was most similar to the target as quickly and accurately as possible. Participants were given 1500 ms to respond with a left or right button press for the corresponding flanker.

The task included two types of trials: switch trials and non‐switch/stay trials. During switch trials, the flankers to which the participants were discriminating the targets from changed, that is, there was a switch in the task rules. These events were indicated by the presentation of the warning red rectangles and fixation cross in a new location, followed by a new set of flankers. During non‐switch trials, the task rules, that is, the relevant flankers, remained unchanged and only a red fixation cross (500 ms) preceded the appearance of a new target image.

### 2.3 Task parameters and experiment space

The parameters of the cognitive paradigm were modified within the predictability along three dimensions: sequential, spatial, and perceptual predictability of switch events. These dimensions were limited to a number (three) that allowed the experiment space to be sampled in a single session, and the three dimensions were selected based on previous work showing modulations in sequential (Ruge et al., 2013), spatial (Vallesi et al., 2015), and perceptual (Crittenden et al., 2015; Smith et al., 2018) dimensions of switching recruited distinct patterns of brain activity. The sequential aspect referred to the frequency at which switch events occurred; the spatial component was linked to the location of the new relevant stimuli; and the perceptual aspect alluded to the nature of the incoming relevant stimuli, that is, the category of the rules (faces, abstract lines, abstract lines, or rooms).

A 2 × 2 × 2 experiment space was constructed by assigning to each sub‐dimension two levels of complexity; in other words, the 2 × 2 × 2 space can be represented as sequentially predictable and unpredictable × perceptually predictable and unpredictable × spatially predictable and unpredictable conditions. The switch events in the first level were characterised by having a highly predictable sequence: sequentially there was a change in rules every four trials, with every switch the location of the stimuli shifted one position downwards, and perceptually the stimuli changed from faces to abstract lines to rooms to abstract figures. The switches in the second, more difficult, less predictable level followed a pseudo‐random sequence: sequentially a switch could occur at any trial within a grouping of four trials (keeping the total number of switches constant), spatially the relevant stimuli could switch one position upwards or downwards (maintaining the frequency of direction a maximum of two switches) and perceptually the category of the stimuli was randomly chosen (ensuring it did not follow the predictable sequence and it was distinct from the preceding rule).

**FIGURE 1:**
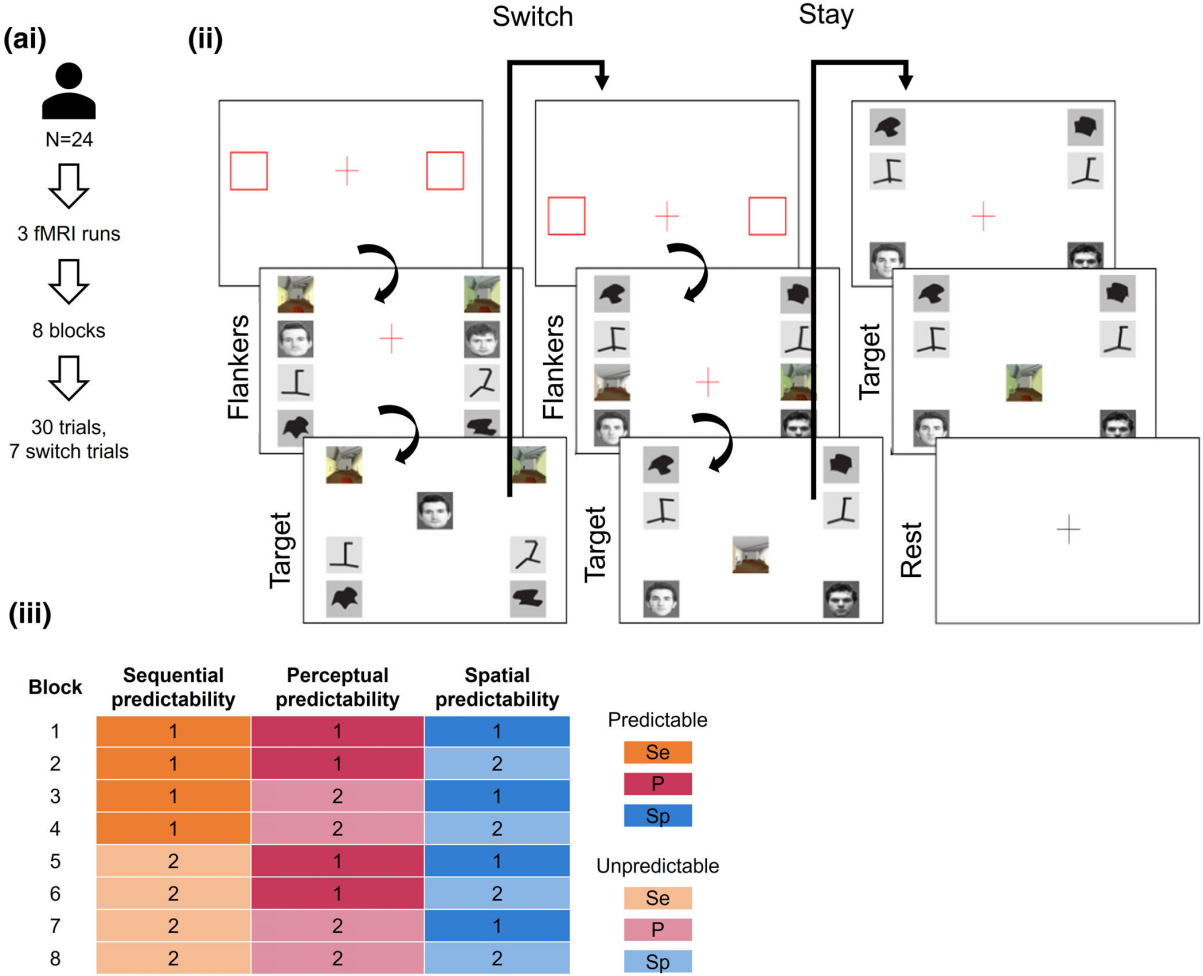
(ai) Participants performed three runs in the MRI scanner. Each run consisted of 8 blocks of the task with 30 trials per block, of which 7 were switch trials. (ii). On the first trial and every switch trial, the participant was first presented with a slide indicating where their attention should be directed (red squares and red fixation cross, 0.5 s). They were then presented with flanker images (four pairs of left and right stimuli, same stimuli category per row) and a red fixation cross presented at the level where their attention should be directed (Flankers, 4 s). This was followed by a target slide, where a probe image replaced the red fixation cross, and participants performed a simple binary‐discrimination task, by matching the probe to one of the flanker stimuli according to which of the flankers was most similar to the target (1.5 s). Stay trials followed the same pattern with the difference that after the target slide, the nonrelevant flankers appeared on the screen alongside a red fixation cross warning the participant of the subsequent appearance of a new target slide. (iii) The combinations of dimensional levels used to define the parameters of each task block with the corresponding colour schemes represented to the right. Trial predictability was modulated along the following three dimensions: the frequency of occurrence of switch events (sequential predictability), the category of the incoming stimuli (perceptual predictability), and the location of the new relevant flanker images (spatial predictability). The order of the blocks was randomised for every run and participant.

The size of the experiment space allowed for a full‐factorial experiment to be conducted, where every possible combination of dimensions was sampled (Figure 1aiii). For example, in a block, the task could have sequential complexity of level 1, spatial complexity of level 2 and perceptual complexity of level 1.

### 2.4 Experimental procedure

The participants performed three back‐to‐back fMRI runs. Within each run, the participants performed eight blocks of 30 trials of the switching task, with 16 s of rest in between. Each block corresponded to one of the previously defined combinations of sequential, perceptual, and spatial predictability, thus, over the entire session the fully factorial design ensured each participant performed 90 trials of every combination of the three switch dimensions. The order of block presentation was randomised across runs and participants. Participants were trained in the task by performing one practice block outside the scanner. In the scanner, the task was projected onto a screen that the participants could see through mirrors placed on the head coil and the responses were recorded using a pair of MRI compatible response grips (ResponseGrip, NordicNeuroLab AS, Bergen, Norway). Participants used the index finger on their right or left hand to select whether the target image was more similar to the right or left flanker of interest, respectively.

### 2.5 fMRI acquisition

MRI data were acquired in a 3 T Siemens Verio (Siemens, Erlangen, Germany) at the Imperial College London Central Imaging Facility using a 32‐channel head coil. Standard T1‐weighted structural images were acquired using an MP‐RAGE sequence, isotropic voxel of 1 mm^3^, repetition time (TR) of 2300 ms, echo time (TE) of 2.98 ms, inversion time of 900 ms, flip angle (FA) of 90°, field of view of 256 × 256 mm, 256 × 256 mm matrix, 160 slices and GRAPPA acceleration factor of 2. For the fMRI images, a T2*‐weighted echo‐planar imaging (EPI) sequence was acquired using an isotropic voxel of 3 mm^3^, TR of 2 s, a TE of 30 ms, FA of 80°, field of view of 192 × 192 × 105 mm, 64 × 64 matrix, 35 slices, and GRAPPA acceleration factor of 2.

### 2.6 fMRI preprocessing

The fMRI Expert Analysis Tool (FEAT, Version 6.00) from the FMRIB's Software Library (FSL) (Jenkinson et al., 2012; Smith, 2004) was employed for the preprocessing and analysis of the fMRI data. FMRIB's Linear Image Registration Tool (Jenkinson et al., 2002; Jenkinson et al., 2012) was used to register the extracted brain tissue (Smith et al., 2012) to the Montreal Neurological Institute (MNI) standard atlas. Head motion was estimated using FSL motion outliers through DVARS (the spatial root mean square of the data after temporal differencing) (Power et al., 2012). No participants met criterion for exclusion due to excessive motion (DVARS >50 in more than 20% of the volumes).

Motion‐correction of the images was performed with MCFLIRT (Jenkinson et al., 2002), and spatially smoothed with a 5 mm Gaussian kernel filter. A temporal high‐pass filter with a cut‐off of 100 s (Gaussian‐weighted least‐squares straight line fitting) was applied to remove low frequency artefacts. The EPI sequences were registered to the MNI space using the T1‐weighted images as intermediate by first carrying boundary‐based registration (Greve & Fischl, 2009) to the main structural image followed by affine registration to the standard brain space (Jenkinson et al., 2012; Jenkinson & Smith, 2001).

### 2.7 fMRI task analysis

#### 2.7.1 Task modelling

FEAT was used to analyse the preprocessed fMRI data. General linear models (GLMs) were constructed with regressors corresponding to task blocks (all trials included in a block) and switch trials (only trials where new rules were presented) for each of the conditions tested (the eight combinations of dimensions). Thus, 16 regressors of interest were fitted into each subject‐level GLM. The task regressors were modelled by convolving a double‐gamma haemodynamic response function (HRF) with a boxcar kernel.

Movement‐related noise was accounted for by adding 24 motion regressors (translation and rotation in three directions, the square of the six motion parameters and their temporal derivatives) to the design matrix. Additional regressors of no interest were added to the GLM as nuisance regressors and used for censoring of motion outliers (as estimated through the fsl_motion_outliers tool using DVARS with a threshold of 50%).

The following contrasts of interest were generated: all task blocks (“Task > Rest”) and all switch events (“Switch > Rest”). A second‐level analysis using a fixed effects model was performed to estimate each subject's mean response across the three runs. This was done by forcing the random effects variance to zero in FMRIB's Local Analysis of Mixed Effects (FLAME) (Beckmann et al., 2003; Woolrich et al., 2004) for each subject‐level contrast. The resulting contrast values and variances were fed into a third‐level analysis to combine data from all participants for the relevant contrasts using FLAME 1 (Beckmann et al., 2003; Woolrich et al., 2004). A Gaussian random‐field based cluster inference (threshold of *z* > 3.1 and cluster‐correction significance threshold of *p* < .05) was applied to threshold the final Z statistical images.

#### 2.7.2 Switch dimension modelling

GLMs were constructed with regressors corresponding to either switch or stay trials for each of the conditions tested. Eight regressors of interest were fitted into each subject‐level GLM. The task regressors were modelled by convolving a double‐gamma HRF with a boxcar kernel. Movement‐related noise was accounted for by adding 24 motion regressors to the design matrix. Contrasts of interest for each dimension were generated. The contrasts were “Sequentially predictable > Unpredictable,” “Perceptually predictable > Unpredictable,” and “Spatially predictable > Unpredictable” and their converses.

A second‐level analysis using a fixed effects model was performed to estimate each subject's mean response across the three runs. This was done by forcing the random effects variance to zero in FLAME (Beckmann et al., 2003; Woolrich et al., 2004) for each subject‐level contrast. The resulting contrast values and variances were fed into a third‐level analysis to combine data from all participants for the relevant contrasts using FLAME. The resulting Z statistical images were thresholded using Gaussian random‐field cluster inference (initial voxel‐level threshold of *z* > 3.1 and cluster‐correction at *p* < .05).

#### 2.7.3 Parcellation of activity maps

For each contrast from task and switch dimension modelling, voxels showing significant activity were parcellated into regions of interest (ROIs) using a three‐dimensional watershed algorithm from the Function and Structural Integration of Neuroimages (Fusion‐WS) toolbox (Daws et al., 2022). The algorithm works by inverting and ranking the input activation map, which creates a topographical representation with peak voxels at the bottom of a “valley.” These valleys are then “flooded,” starting with a radius of five voxels. Valleys are continuously flooded at a steady rate, with “water” filling the basins from the bottom, that is, the lowest‐ranked voxel. Each voxel can have one of the following outcomes as flooding progresses: first, if a voxel is within the radius of the voxel at the lowest point in the basin, they are assigned the same label. Second, if a voxel has no neighbours, it is assigned a unique label. Third, if a voxel has multiple neighbours, a “winner takes all” approach is used to label to a voxel. Finally, if neighbouring labels are below a merge threshold of 100 voxels, they are merged. All labelled basins can then be considered parcellated regions from the continuous activity map.

The watershed algorithm's “label to table” function then assigns structural and functional labels to the regions. For example, if a region is predominantly in the left insula as defined by the anatomical automatic labelling atlas (Yeo et al., 2011), it will be assigned that label, as well as the label of ventral attention network, since the left insula is within the Yeo et al‐defined ventral attention network. Thus, the continuous activity map is parcellated, with regions ascribed an atlas‐defined functional and anatomical labels.

#### 2.7.4 Computing mutual information functional connectivity

**FIGURE 2:**
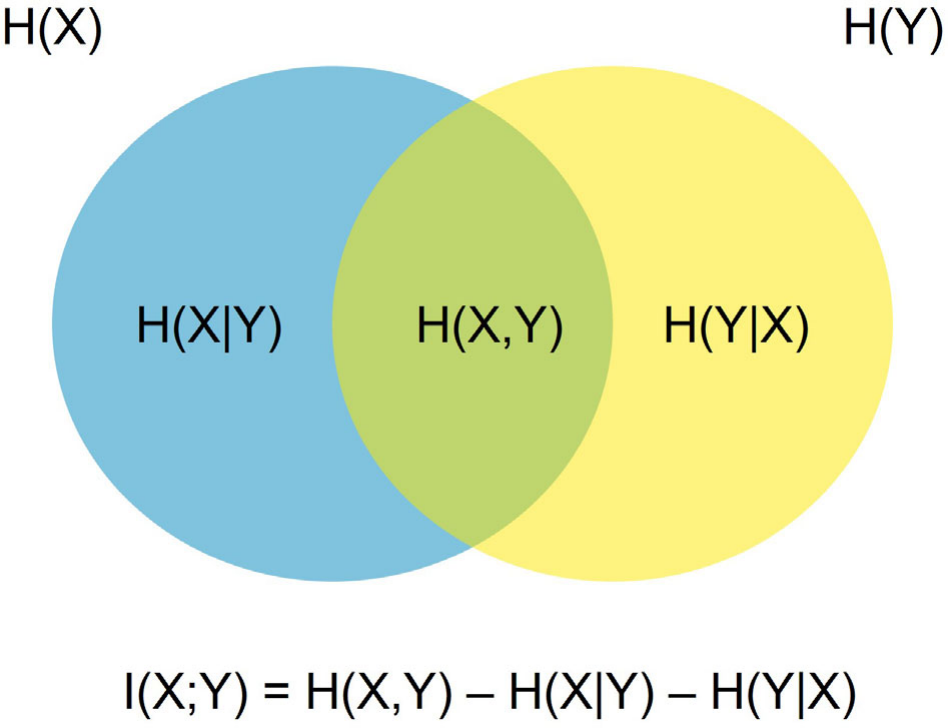
Depiction of the mutual information of the two variables X and Y. H(X) or H(Y) is the (marginal) entropy of each variable, whereas H(X|Y) or H(Y|X) is the conditional entropy (the entropy that X will occur given Y). The area where the conditional entropies overlap, in green, is the joint entropy (i.e., likelihood of co‐occurrence) of each variable. The mutual information of X and Y is computed as the joint entropy of X and Y minus the conditional entropies of X and Y given Y and X, respectively.

We used mutual information as a non‐parametric assessment of dependence between two signals, which serves as a measure of functional connectivity (Afshin‐Pour et al., 2011; Lizier et al., 2011; Vergara et al., 2017; Wang et al., 2015). Mutual information functional connectivity (MiFC) has an advantage over parametric measures of pairwise connectivity, such as Pearson correlations, as it captures nonlinear, causal relationships between two regions (Wang et al., 2015). For each participant, for each run, the 100‐parcel, 17‐network Schaefer atlas (Schaefer et al., 2018; Yeo et al., 2011) was registered to participant space, and BOLD timeseries were extracted from each region. The timeseries were z‐scored to centre mean 0 and ±1 SD. To account for the lag between task conditions and haemodynamic response, we shifted the timeseries forward 10 s (five volumes). We also computed results for the timeseries shifted forward 8 and 12 s (four and six frames, respectively) to ensure results were not confounded by the method of accounting for the HRF. We observed that when the timeseries is shifted forward 8 or 12 s the results were in line with those described in this work (Supplementary Material Figures 2 and 3). The pairwise mutual information for all ROIs was computed using algorithms from Peng et al. (2005), which enables the computation of mutual information between continuous signals. Mutual information captures how well the state of one variable informs the state of another, that is, how observing one variable reduces the entropy of another. Entropy represents the “uncertainty” or “information” contained in an event, where the more probable/predictable an event is, the lower its entropy. A typical example can be found in a coin toss—given the random (i.e., highly uncertain) nature of a coin toss, the entropy of the outcome of a coin toss is maximal. Another example can be found in fMRI data, in the entropy of observing an increase in the magnitude of the BOLD signal in a brain region—for example, the posterior cingulate cortex (PCC). One way to reduce the entropy of such an event would be to collect fMRI data during a resting state study. Therefore, the predictability of PCC activity would be contingent on whether data were collected during a particular condition. This is considered the *conditional entropy*, that is, the entropy that an event will occur given a particular condition. Additional variables and the information they provide can be included; for example, knowing the presence and amount of motion artefacts also reduces in uncertainty about whether the activity in the PCC has increased. The *joint entropy* of observing activity in the PCC can thus be the product of considering the conditional entropy of both the condition in which data were collected, and the motion of participants during data collection. *Mutual information* is computed from both the joint and conditional entropy and conveys whether observing the condition of one variable reduces the uncertainty (entropy) in another variable (Figure 2). In the context of this work, we compute the mutual information of two regional timeseries to capture how well observing the state of one region predicts the state of another region.

Mutual information for continuous variables *x* and *y* (as applied here, *x* and *y* are timeseries of BOLD intensity from two different regions) is canonically defined as

(1) $$I\left(x;y\right)=\iint p\left(x,y\right)\log\frac{p\left(x,y\right)}{p\left(x\right)p\left(y\right)}\text{dxdy},$$

where *p*(*x*), *p*(*y*), and *p*(*x*,*y*) are probabilistic density functions (Afshin‐Pour et al., 2011; Peng et al., 2005; Wang et al., 2015). Because *x* and *y* have a limited number of samples, Peng et al. used a kernel density estimation in line with previous work (Kwak & Choi, 2002) to approximate *I*(*x*;*y*) as $\hat{p}\left(x\right)$ in the following manner:

(2) $$\hat{p}\left(x\right)=\frac{1}{N}\sum_{i=1}^{N}\delta \left(x-x^{\left(i\right)},h\right).$$

In Equation (2), $\delta \left(.\right)$ is a Gaussian kernel density window function, *x* ^*i*^ is the *i*th sample, and *h* is the window width, which scales with *x*. It is necessary that *h* scales with *x*, as one of the conditions for generating a density estimation is that the probability of the occurrence of *x* out of the total population equals 1—thus, as the size of *x* increases, so too must *h* (Sneller, 2017).

A Gaussian kernel density means that $\hat{p}\left(x\right)$ can be computed using

(3) δz,h=exp−zT∑−1z2h2/{2πd2hd∑12},

where *z* = *x‐x* ^(*i*)^, ∑ is the covariance of *z*, and *d* = 2 (for the bivariate variables *x* and *y*). When *d* = 2, as is our case, $\delta \left(.\right)$ Equation (2) returns the density estimation $\hat{p}\left(x\right)$, of the bivariate variable (*x*,*y*), *p*(*x*,*y*), which is also the joint density of *x* and *y*. After computing the joint probability distribution using Equation (2), we then computed the mutual information between variables *x* and *y* in Equation (1). Computing the pairwise mutual information among all timeseries generated a 100‐by‐100 miFC matrix, where each cell was valued between 0 (indicating the timeseries were not related) and 1 (indicating an entirely dependent relationship between timeseries).

#### 2.7.5 Within‐ and cross‐participant reliability in miFC

We used the intraclass correlation coefficient (ICC) to evaluate the reliability in miFC per region within and across participants. In line with Koo and Li (2016), we computed the ICC for a two‐way model, with absolute agreement and mean ratings. ICCs were computed for each region across runs to assess within‐subject reliability, and ICCs for each region across all participants and runs were computed to assess across‐subject reliability in miFC. The ICC score for the miFC for all ROIs across the three runs showed good reliability within subjects (mean = 0.83, std = ±0.11) (Supplementary Material Figure 1ai); however, one participant exhibited noticeably less reliability in miFC for all regions across runs (mean = 0.52, std = ±0.09), and was excluded from further miFC analyses. After removal, the mean within‐subject reliability improved (mean = 0.85, std = ±0.09) across all subjects (*n* = 22). The reliability of all regions across participants per run was moderate (mean = 0.60, std = ±0.07) (Supplementary Material Figure 1bi). The lower interindividual reliability as compared to within individual reliability is expected (McGonigle, 2012; Savoy, 2006).

#### 2.7.6 Assessing the effect of switch dimensions on miFC

We evaluated the effect of switch dimensions on miFC by first computing the average miFC among all ROIs in the 100‐parcel, 17‐network Schaefer atlas (Schaefer et al., 2018) across runs for each block, for each participant (as in Lizier et al., 2011). We employed the Schaefer atlas because Schaefer‐defined regions are associated with one out of the 7 or 17 Yeo et al. resting state networks. This offers a common functional network to relate results between the activity‐based, watershed‐defined ROIs and the atlas‐defined ROIs. Put another way, if a region from one network was significantly influenced by a switch dimension as determined by the activity‐based analyses, and there is a substantial influence of the same switch dimension in the same network's connectivity as defined by miFC analyses, then we could more confidently conclude there is a relationship between that network and switch dimension.

We used a full factorial 2 × 2 × 2 repeated measures ANOVA with the three predictability dimensions as factors (sequential, perceptual, and spatial) to assess whether there was an effect of switch dimensions on miFC. Results were FDR‐corrected for the number of comparisons in miFC. Directions of effects were computed using Wilcoxon sign‐rank tests of miFC between the two difficulty levels per dimension.

We then investigated how the significant changes in pairwise miFC were distributed across functional networks. We grouped regions according to the functional network in which they reside and defined each network as a node. Nodes were connected by edges if the miFC between the nodes had a significant change in miFC due to sequential or perceptual predictability. For example, the precuneus and inferior parietal lobule are both a part of the DMN network/node, and a significant change in miFC between the inferior parietal lobule and the precuneus or motor cortex would count as a within‐ or between‐network change in connectivity, or edge. Edges were not counted twice, for example, a significant change in miFC between the inferior parietal lobule and precuneus would only be counted once. We also computed the total number of edges that emanated from each node, referred to as the node's degree. Edges were weighted by the number of regions within each network showing increases in miFC. For example, if two regions from the control network have significantly increased miFC to two regions within the DMN, the edge weight between the control network and DMN would be 2.

### 2.8 Statistical analyses

**FIGURE 3:**
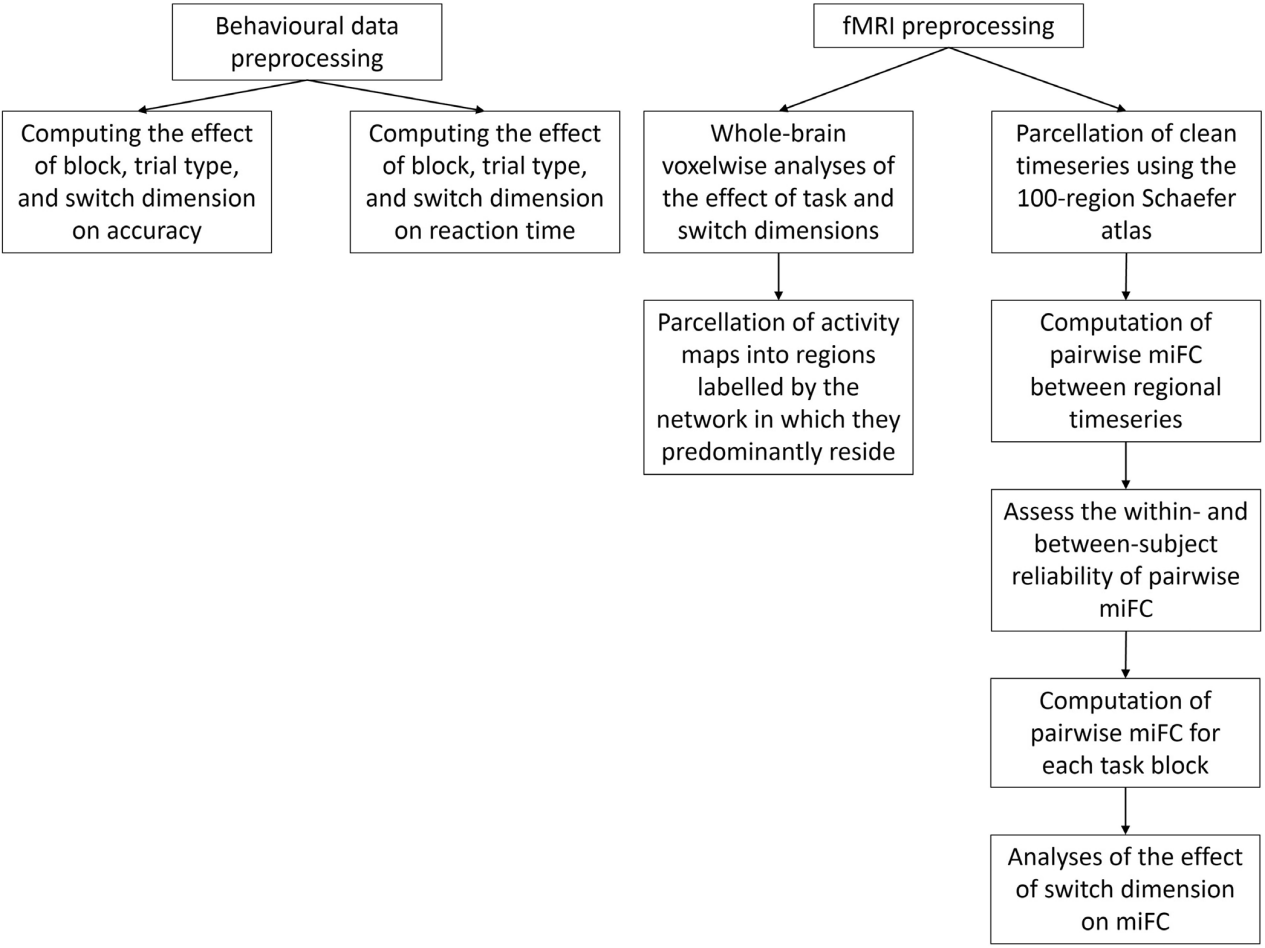
Schematic of analyses pipeline for behavioural and neuroimaging data.

Statistical analyses were performed using MATLAB 2021a. Raw accuracy, defined as the number of correct responses versus misses and incorrect responses, and correct raw RTs from correct switch and stay trials were computed, normalized through rank inverse transform as in Jolly et al. (2020), and analysed using linear mixed effects models (LMEM). The influence of block and trial as fixed effects on correct, normalised RT were assessed using LMEM with a normal distribution and subjects as random effects. The influence of each switch dimension and the interactive effect of switch dimension and trial type (switch or stay) on RT was assessed with an LMEM where each dimension was a fixed effect, using a normal distribution, and subjects as random effects. We computed the accuracy for switch and stay trials and assessed the influence of block and trial type using an LMEM with a binomial distribution, with block and trial type as fixed effects and subject as random effects. The influence of each switch dimension on accuracy, as well as the interactive effect of switch dimension and trial type, was assessed with an LMEM with a binomial distribution, where each dimension was a fixed effect, and subjects were included as random effects. Significance for all models was set at *p* < .05 (Figure 3).

## 3 RESULTS

### 3.1 Behavioural performance

**FIGURE 4:**
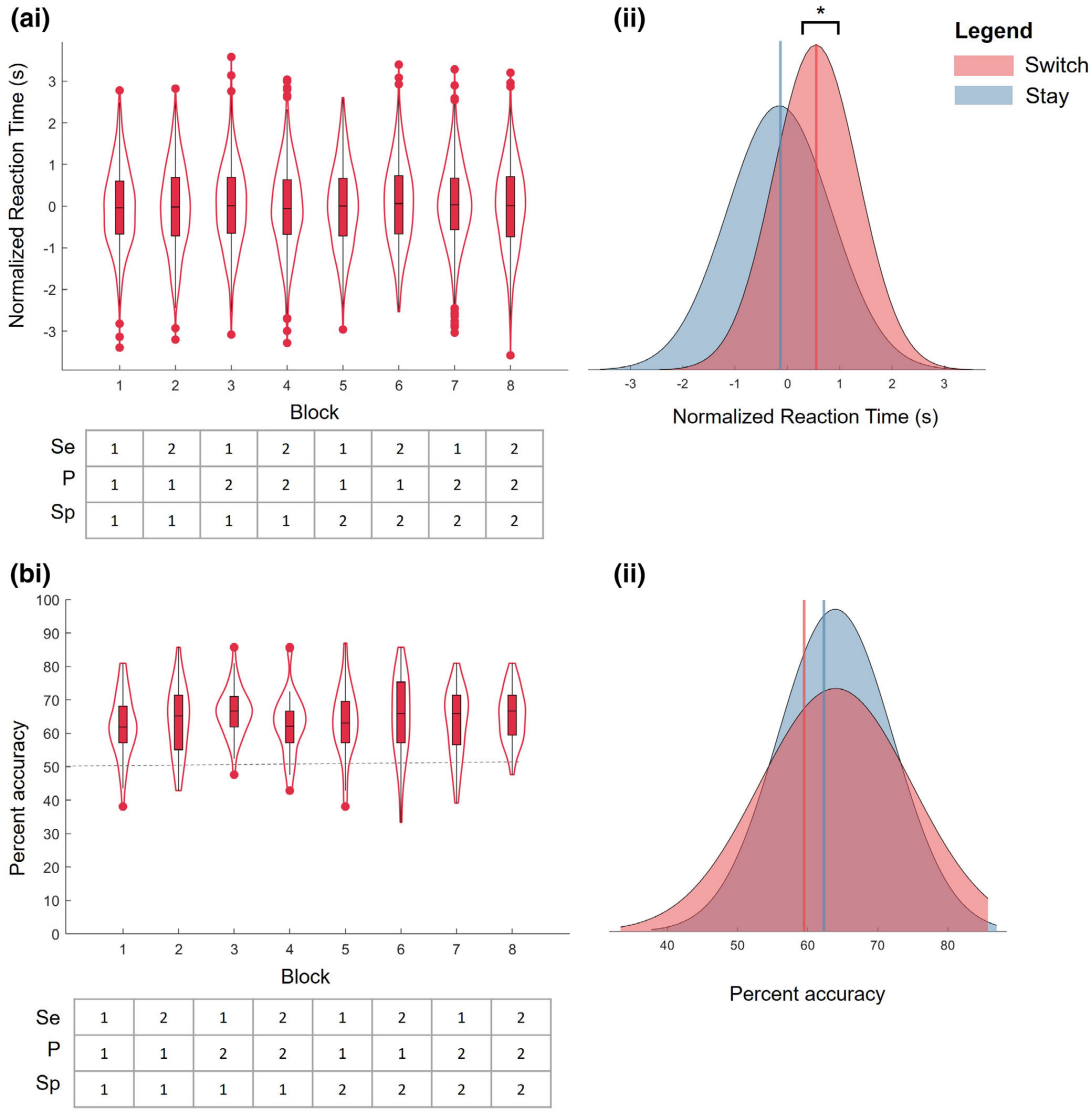
(ai) Violin plots of normalized reaction time (RT) and (bi) percent accuracy per block. The top and bottom edges of the boxes represent the 25th and 75th percentiles, with the median shown by the horizontal black line. Extension of the whiskers indicates 1.5 the interquartile range, and outlier performance is indicated by individual data points. A kernel density estimate of the data provides the edges to the violin plot. The grey dashed line shows chance performance. The tables below the x‐axis indicate the level of each switch dimension per block; Se, sequential predictability; P, perceptual predictability; Sp, spatial predictability. Distributions of (bii). Normalized RT and (bii) percent accuracy for switch and stay trials, with lines indicating the mean of each distribution. * indicates significant difference where *p* < .05.

There was a significant, main effect of trial type on RT (*F*(1,5855) = 151, *p* = 3.4e‐22) where switch trials showed longer RT than stay trials (Figure 4aii). There was not a significant main effect of block (*F*(7,5855) = 0.85, *p* = .55) or an interactive effect of block and trial (*F*(7,5867) = 0.41, *p* = .90) on RT. There was no significant effect of sequential (*F*(1,5867) = 1.3, *p* = .25), perceptual (*F*(1,5867) = 0.15, *p* = .69), or spatial (*F*(1,5867) = 0.32, *p* = .57) predictability on RT, nor were there any interactive effects of sequential (*F*(1,5867) = 0.52, *p* = .47), spatial (*F*(1,5867) = 0.35, *p* = .55), or perceptual predictability (*F*(1,5867) = 0.18, *p* = .67), and trial type on RT.

Participants performed the task well, and accuracy was significantly above chance (*t*(35998) = 32, *p* = 5.7e‐223, *D* = 0.34). There was not a significant main effect of block (*F*(7,17,984) = 1.8, *p* = .07) or trial type (*F*(1,17,984) = 0.89, *p* = .34) or an interactive effect between block and trial type (*F*(7,17,984) = 0.78, *p* = .61) on accuracy (Figure 4b). There was a significant main effect of sequential predictability (*F*(1,17,996) = 4.6, *p* = .03) on accuracy, with higher accuracy during more sequentially predictable trials. There were no significant effects of spatial (*F*(1,17,996) = 0.49, *p* = .48) or perceptual predictability (*F*(1,17,996) = 0.49, *p* = .48), and no interactive effects of sequential (*F*(1,17,996) = 1.8, *p* = .18), spatial (*F*(1,7996) = 2.2, *p* = .14), or perceptual predictability (*F*(1,17,996) = 0.10, *p* = .78) and trial type on accuracy.

Therefore, the task produced the expected effects on RTs whilst maintaining a suitable level of accuracy. Sequential predictability was the only switch dimension that showed a significant effect on task performance.

### 3.2 Switching recruited broad activity patterns that included resting state networks

**FIGURE 5:**
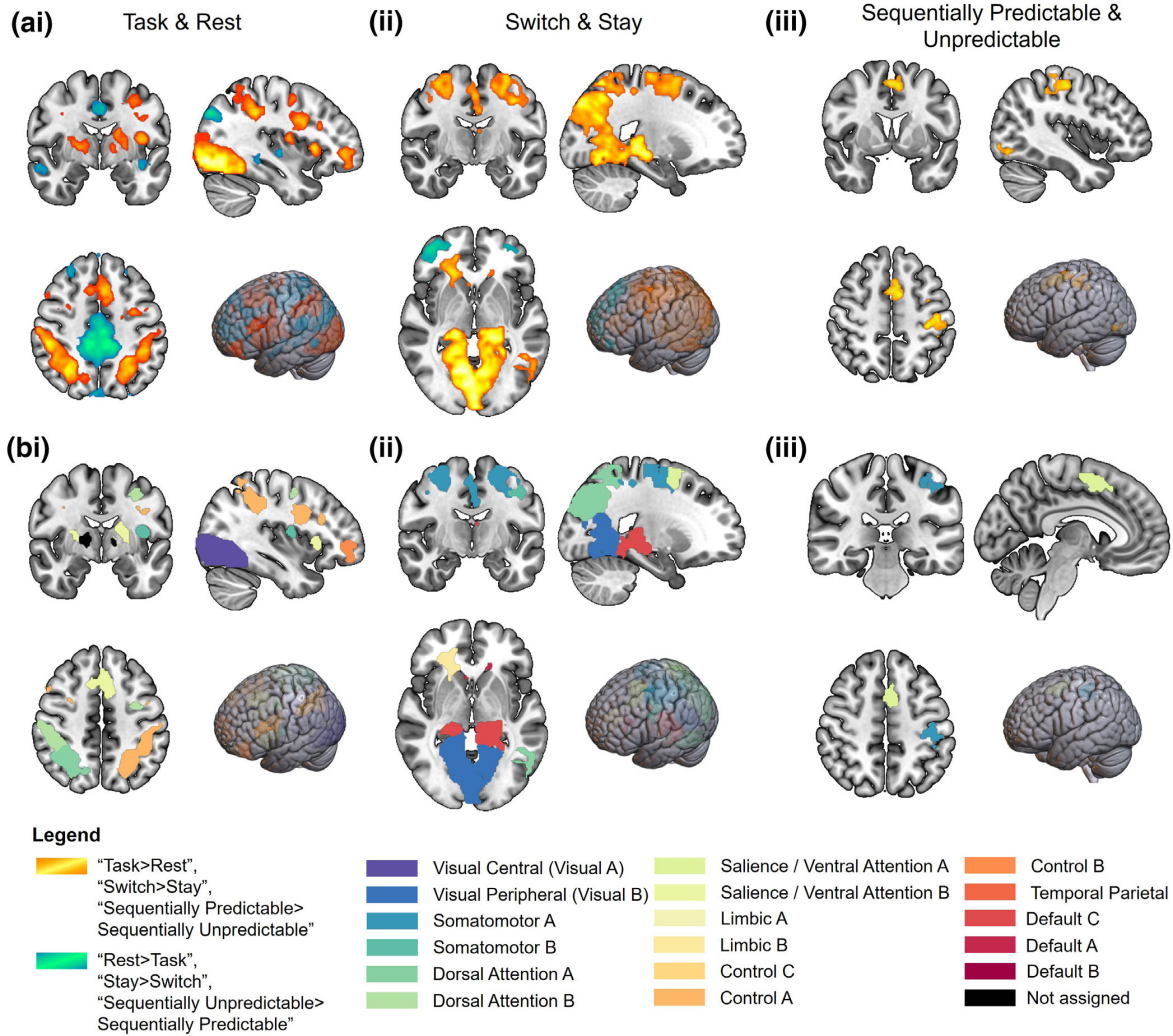
Whole‐brain activation masks for the contrasts (ai) “Task > Rest” and “Rest > Task,” (ii) “Switch > Stay” and “Stay > Switch,” and (iii). “Sequentially Predictable > Sequentially Unpredictable.” Activity maps from the “Task > Rest,” “Switch > Stay,” and “Sequentially Predictable > Sequentially Unpredictable” contrasts are in warm colours, whereas “Rest > Task,” “Stay > Switch,” and “Sequentially Unpredictable > Sequentially Predictable” are in cool colours. Results from the voxelwise analysis were cluster corrected (*Z* > 3.1, and *p* < .05) and were overlaid on a smoothed MNI152 brain template using MRIcroGL. Activation maps for the contrasts (bi) “Task > Rest,” (ii) “Switch > Stay,” and (iii). “Sequentially predictable trials > sequentially unpredictable trials” were parcellated by the Fusion‐WS toolbox (Daws et al., 2022) and regions of interest (ROIs) were coloured according to the Yeo 17 network in which they reside.

The contrast “Task > Rest” showed regions of activity in the visual A, somatomotor B, dorsal attention A and B, salience/ventral attention B, limbic B, control A and B networks (Figure 5ai). One region was in the right thalamus and was not within any Schaefer networks. The contrast “Switch > Stay” rendered a broad activation pattern (Figure 5aii), with activity observed in visual B, somatomotor A and B, dorsal attention A and B, salience/ventral attention A, limbic B, default A and C networks (Figure 5bii). Only the “Sequentially Predictable > Sequentially Unpredictable” contrast showed significant differences within a switch dimension (Figure 5aiii), with regions active within somatomotor A and salience/ventral attention A networks (Figure 5biii).

While the task showed expected activation within the MDC, the “Switch > Stay” contrast revealed that the more cognitively demanding switch condition recruited a broader activation pattern than the “Task > Rest” contrast. The “Switch > Stay” map not only included expected MDC activation, but also included posterior and medial regions within the DMN. We also observed that the “Sequentially Predictable > Sequentially Unpredictable” contrast showed significant changes in activity in somatomotor and attentional networks, but no other dimension‐specific contrasts showed significant changes in the magnitude of the BOLD signal.

### 3.3 Sequential and perceptual predictability influence miFC

In the previous section, we characterised brain activity in response to task and observed differences in brain activity in response to switch or sequentially predicable trials. This section explores whether there are differences in functional connectivity in response to switch dimensions. MiFC is employed as a metric of functional connectivity, since mutual information captures the nonlinear dependence between two regions by quantifying how much information is shared between regions (Li, 2022; Shannon, 1948).

**FIGURE 6:**
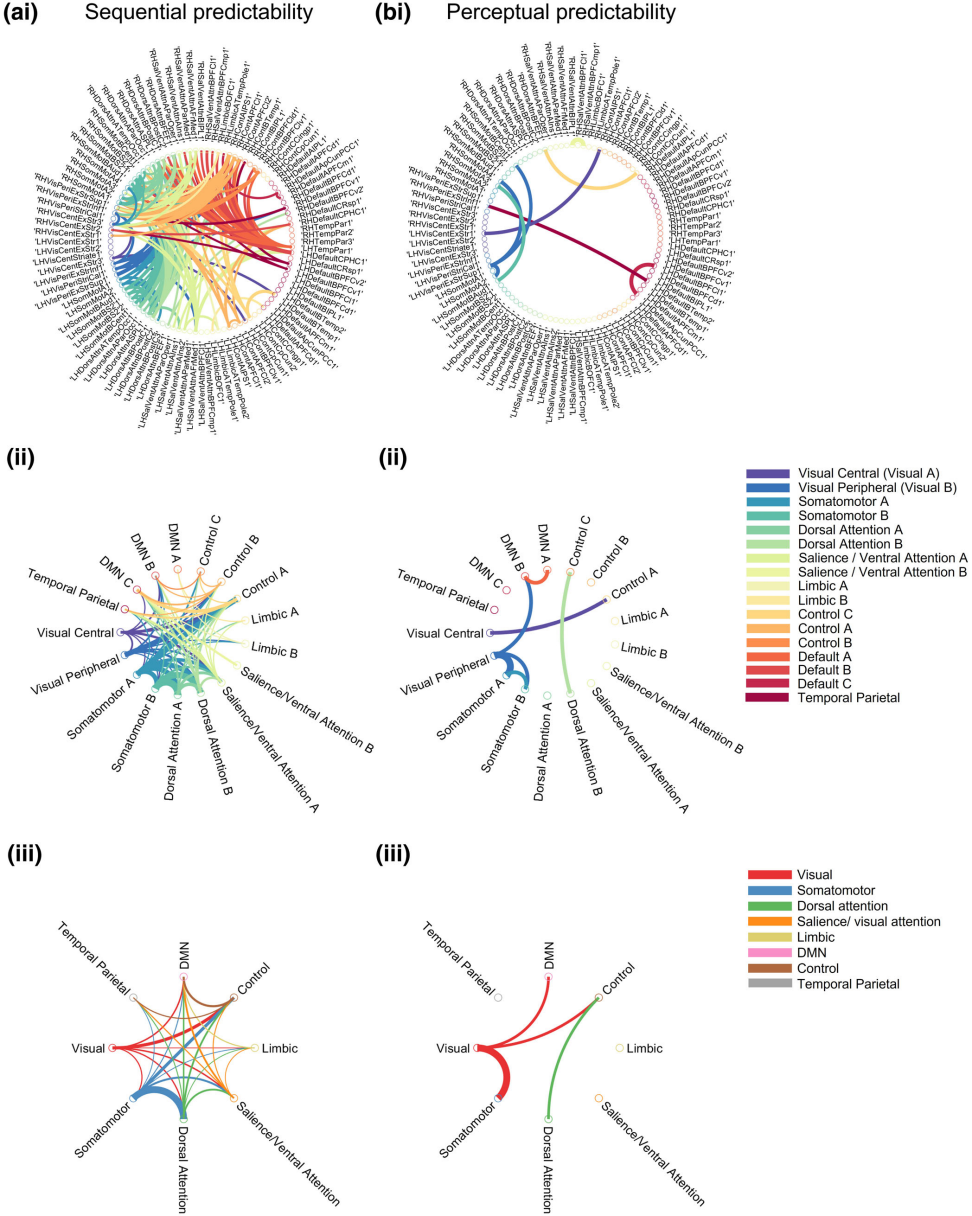
There were significant effects of (a) sequential (b) and perceptual predictability on mutual information functional connectivity (miFC). Regions whose miFC was significantly influenced by (ai) sequential and (bi) perceptual predictability after FDR correction for multiple comparisons were connected by edges. The edge's width was scaled to the effect size (*η* ^2^). The first region out of the pair determines the colour of the connection, and colour is assigned according to the network the first region is in. Regions with significant changes in miFC due to (aii) sequential and (bii) perceptual predictability are grouped by network, where each node represents a network. The edges are scaled with their weight, which captures the proportion of connections between two connected network and the total number of edges. To aid visualization of networks with miFC influenced by (aiii) sequential (biii) perceptual predictability, we created graphs that grouped subnetworks together. For example, control networks A, B, and C were consolidated into one control network. Networks with a degree of 0 or within‐network increased in miFC are not included in the network connectivity graphs.

We observed significant changes in miFC due to sequential (Figure 6a) and perceptual (Figure 6b) predictability, but not spatial predictability. There were 182 pairs of regions with significant effects of sequential predictability on miFC, of which 98% showed that increases in sequential predictability were associated with increases in miFC (Supplementary Material Table 1). In contrast, 7 out of 10 (i.e., 70%) of the significant effects of perceptual predictability showed a negative relationship between perceptual predictability and miFC (Supplementary Material Table 2).

We found that higher sequential predictability increased miFC between the DMN and subnetworks within the MDC (somatomotor, attention, and control, and visual networks) (Figure 6aii) (Supplementary Material Table 3). The somatomotor networks, followed closely by the dorsal attention networks, showed the greatest increase in degree due to higher sequential predictability (degree of 91 and 81, respectively) (Supplementary Material Table 4). These increases in degree largely connected the somatomotor and dorsal attention networks to each other (Figure 6aii,iii). Other networks showing notable increases in degree due to higher sequential predictability included the DMN, control, visual, and salience/ventral attention networks (Supplementary Material Table 4). The DMN shows changes in regions within DMN A, B, and C networks, showing sequential predictability influences miFC in anterior, medial, and posterior portions of the DMN.

In addition, there was a generalised increase in cross‐network degree (Figure 6aii,iii).

Shifting focus from a network‐level perspective towards a regional focus, we note that the majority (63%) of sequential predictability‐related changes in miFC in DMN regions are within the parahippocampal cortex (PHC). The PHC showed increased miFC to MDC regions including the anterior insula, iPS, and dlPFC. Another DMN region, the ventral PFC, also showed increased miFC to a region within the MDC, the pre‐SMA. Thus, miFC between regions within the MDC and DMN showed a positive relationship with sequential predictability.

Between‐network changes in miFC due to perceptual predictability were less pronounced, but involved decreases in miFC between the MDC and DMN when perceptual predictability increased (Figure 6bii) (Supplementary Material Table 5). The largest decreases in degree were seen in the DMN, visual, and somatomotor networks, which all decreased their degree by 5 (Supplementary Material Table 6). These decreases in degree largely separated the visual network from the somatomotor, DMN, and control network (Figure 6aii,iii).

In summary, sequential and perceptual predictability differentially influenced connectivity, where increases in sequential predictability recruited connectivity between somatomotor, control, DMN, and other networks, whereas increases in perceptual predictability reduced connections among the same networks.

## 4 DISCUSSION

Here, we report results of a novel fMRI switching task where imaging results showed concurrent activation of the DMN and MDC during switch trials as compared to stay trials. This concomitant activation inverts the stereotypical association of task difficulty with MDC and DMN network activity (Buckner & DiNicola, 2019; Fox et al., 2005; Fransson, 2005). Early evidence from event‐related fMRI studies strongly supported a “task‐negative” activation profile for the DMN, with the popular interpretation being that this reflected a role in internally generated cognition and disengagement from external demands (Raichle & Snyder, 2007). Numerous experiments reported deactivation of the DMN during task relative to rest, and the level of deactivation has been observed to scale with the general cognitive difficulty of the task (Mayer et al., 2010; McKiernan et al., 2003). Further evidence has come from studies where higher DMN correlation was observed during rest than task (Fox et al., 2005; Raichle & Snyder, 2007).

The last few years have seen a diversification in perspectives on the functional role of the DMN in cognition (Buckner & DiNicola, 2019; Margulies & Smallwood, 2017). For example, it has been reported that the DMN reconfigures its internal functional connectivity state when a person is engaged in a task (Vatansever et al., 2015; Vatansever et al., 2016), and modulates its activity according to experimental demands (Koshino et al., 2011, 2014; Spreng & Andrews‐Hanna, 2015). Most relevantly, activation of regions in the DMN have been reported to increase when switches are performed between distinct tasks (Crittenden et al., 2015; Smith et al., 2018). More recent evidence has suggested that the DMN and the MDC serve complementary roles in paradigms where multi‐step decision making takes place, where the DMN tracks shifts between tasks whereas the MDC primarily engages in the steps necessary to complete each task (Wen et al., 2020). Our results accord with this complementary contextualization of the DMN and MDC. We show DMN activity during switch trials as compared to stay trials Moreover, we show increases in miFC between the MDC and DMN during sequentially predictable switches, indicating that the DMN may have a role in anticipating when a task switch will occur. This is also supported by work showing that as a task becomes automated through practice (e.g., when applying learned rules), there is a concomitant increase in event related DMN activity (Hampshire et al., 2016; Hampshire et al., 2019; Vatansever et al., 2017). Automation implies the order of steps needed to complete a task are predictable, and the increase in miFC between the DMN and MDC during sequentially predictable switches supports the possibility that the brain may reconfigure its connectivity to generate a schema of an upcoming task switch. Indeed, research has showed co‐activation of the DMN and MDC regions during predictable task sequences (Bor et al., 2003; Dreher et al., 2002). Work by Bor et al. (2003) investigated the neural processes associated with predictable and unpredictable task sequences and showed unique increases in the magnitude of the BOLD signal in several DMN (PHC, prefrontal cortex) and task‐active (temporal, dlPFC, lateral prefrontal cortex, and inferior parietal lobule) regions that also showed increased miFC due to increased sequential predictability. Bor et al. (2003) suggested these regions subserve a working memory strategy encouraged by the predictable task sequences referred to as “chunking,” which is when task‐relevant material is cognitively reorganised. Though effortful, the organisation of task‐relevant material into accessible structures substantially improves working memory capacity (Thalmann et al., 2019). Chunking actively organises task‐relevant material to facilitate task performance; similarly, MDC and DMN regions may work in a coordinated manner when predictable task structures can be leveraged to generate schema of the upcoming task switch and thus enhance cognitive performance. This aligns with the notion of a many‐to‐many functional mapping between cognitive processes and functional regions of the brain (Lorenz et al., 2018; Soreq et al., 2019), and how the increased cognitive load of switching requires the optimisation of neural processes. This switch‐tracking optimisation may manifest as a network that recruits regions from core DMN and MDC regions.

Though work by Bor et al. (2003) and others (Wen et al., 2020) corroborates our suggestion that regions from the DMN and MDC may coordinate to generate a schema of upcoming task switches, what roles could the DMN and MDC regions play in a switch‐tracking network? We observed the most sensitive region within the DMN to changes in sequential predictability was the PHC, which composed over half of the significant changes in miFC in DMN regions due to sequential predictability. The PHC is associated with contextual tracking and episodic memory (Aminoff et al., 2013), and in turn, temporal tracking and maintaining temporal context (Davachi & DuBrow, 2015). A review of studies by Wang and Diana (2017) characterising the neural correlates of temporal context observed that the PHC is especially recruited during shorter block durations similar to those used in this work (Konishi et al., 2002; St. Jacques et al., 2008). This corroborates the potential role of the PHC in tracking the temporal context during sequentially, but not spatially or perceptually predictable, switches.

If the PHC is responsible for tracking when a switch will occur, what could be the role of the MDC? The MDC coordinates planning to achieve task‐related goals (Duncan, 2010), and has been shown to reconfigure to accommodate shifting task demands (Woolgar et al., 2011). If DMN regions (particularly the PHC) anticipate an upcoming switch, MDC regions may prepare cognitive resources away from recalling the current set of flankers and prepare to encode features of upcoming, new flankers. This suggestion is supported by the inclusion of the iPS and pre‐SMA in MDC regions with miFC affected by sequential predictability, as previous work has shown these regions are sensitive to encoding new rule presentations (Dumontheil et al., 2011). Moreover, all MDC regions with miFC significantly influenced by sequential predictability have been shown to encode task‐relevant stimuli as required by the task employed in this work (Haynes et al., 2007; Li et al., 2007).

The involvement of both the iPS and pre‐SMA is notable. Previous research implicated the iPS and pre‐SMA in focusing attention with respect to an anticipated, upcoming task, a process known as temporal orienting (Coull et al., 2011; Coull & Nobre, 1998). The role of the iPS and pre‐SMA in temporal orienting is heightened when an upcoming task is temporally predictable (Berchicci et al., 2020; Coull et al., 2016). Moreover, work by Periáñez et al. (2022) showed switch trials related to an increased magnitude of the BOLD signal in the iPS, and when continuous theta burst transcranial magnetic stimulation (cTMS) was delivered to the iPS switch RTs increased. Evidence suggests that cTMS decreases local cortical excitability (Huang et al., 2009; Huang et al., 2011), and therefore suggests that iPS activity is causally linked to switching performance (Periáñez et al., 2022).

The pre‐SMA and iPS are part of the somatomotor and control networks, respectively, both of which are in the top three networks exhibiting the greatest proportional change in miFC as a result of increased sequential predictability (Supplementary Material Table 3), with increased miFC from both networks to the DMN. Thus, our work further reinforces the association of the iPS and SMA with temporal orienting, and that the functional roles associated with MDC and DMN regions are related to the neural processes of generating a task schema. Results from this work suggest that these regions transiently shift from their typical network configurations during sequentially predictable switches and coordinate a preparatory response to an upcoming task switch.

This dimension‐specific, many‐to‐many mapping of brain regions not only supports the likelihood of a switch‐tracking network, but also accords with the differing relationships between control and visual networks due to increased perceptual predictability. As perceptual predictability increases, miFC between control and visual networks decreases, in contrast to the increased miFC between the control and visual networks due to higher sequential predictability. The dissociation between control and visual networks scaling with perceptual predictability is supported by work from Woolgar et al. (2011). They showed that increases in perceptual difficulty led to increases/decreases in MDC/visual activity, respectively. The authors reasoned that when task conditions are easy visual stimuli are represented in the visual cortex, whereas more perceptually difficult conditions shifts the coding of visual stimuli to more cognitively oriented (i.e., MDC) regions, resulting in increased MDC activity (Woolgar et al., 2011). Applying this rationale to our results indicates that perceptually predictable switches do not need the additional cognitive resources recruited from the cognitive networks during less predictable switches, resulting in the decreased miFC between the visual and cognitive networks.

A caveat when comparing our results to the work of Woolgar et al. (2011) and other task‐switching studies is that the tasks used here did not use classic task‐switching designs. Instead, they built on more contemporary IBL paradigms (Cole et al., 2010, 2013; Cole et al., 2016; Hampshire et al., 2016; Hampshire et al., 2019; Ruge & Wolfensteller, 2015). These differ insofar as the switching of rules is driven by the presentation of an instruction slide with explicit pictorial depiction of the rules as opposed to a cue initializing a switch between alternative mappings that are coded within WM. However, our IBL task is analogous to this design in several key ways. The task program must be updated in response to the instruction cue, and the reduction in RT on trials after new rules were presented relative to when rules remained the same is a characteristic feature of switching. Furthermore, we argue that generalisation of results across different task conditions provides stronger information for understanding brain functions.

A further caveat is the heterogeneity in the number of regions contributing to each functional network (mean = 6, STD = 2, min = 2, max = 10). Because some networks are composed of a greater number of regions, it is possible that this may skew the likelihood of the involvement of this network. However, we show that the two networks with greatest changes in degree (the somatomotor and dorsal attention networks), are not the two largest networks (the DMN and salience/ventral attention network). This indicates that the functional role of networks (and the regions that compose them), rather than the number of times they are represented, drives their involvement during sequentially predictable switches.

A final limitation of our study could be the simple experimental design that makes inferences to real‐world situations challenging. However, the simple design allowed disentangling the effects of different task dimensions, and it is a necessary first step to better understand the role of the networks during switching. A straightforward next step is to test whether our results hold in a naturalistic switching task (e.g., movie viewing or virtual reality) where real‐world relevance could be more directly inferred (Finn, 2021). It would also be informative to extend the current paradigm to one where there is a deeper structure to the task schema that is learnt progressively, in order to study the shifting role of MDC and DMN regions across the learning curve. Such a task was employed by Daws et al. (2020), which created a self‐ordered‐search task that allowed participants to manage how they split their time between two cognitive tasks. The authors showed that high performers exhibited more structured task engagement, as well as increased DMN activity during easier, repetitive trials. The utilisation of the DMN during predictable trials presumably freed up cognitive resources for large executive shifts, which were marked by increased frontoparietal engagement, and was potentially mediated by observed increases in connectivity between frontoparietal and subcortical regions (Daws et al., 2020). Additional exploration of the link between adaptive network reconfiguration and behavioural performance may inform how the ability to learn and predict the sequential structure of events translates to one's ability to successfully navigate complex daily life. Future work may examine whether disruption of the network dynamics associated with sequential predictability is evident in clinical populations who are prone to executive deficits, such as people with schizophrenia or bipolar disorder (Ancín et al., 2013).

In summary, our results further extend the known roles of the MDC and DMN, and collectively indicate a more active role for regions included in the DMN during task performance than was commonly believed.

## FUNDING INFORMATION

D.L.K. received funding from the University of Surrey. R.L. received funding from the EPSRC (P70597) and the Wellcome Trust (209139/Z/17/Z). I.R.V. received funding from the BBSRC (Ref: BB/S008314/1). A.H. is supported by the Dementia Research Institute and the Imperial NIHR Biomedical Research Centre. The funders had no role in study design, data collection and interpretation, or the decision to submit the work for publication.

## Supporting information


**DATA S1** Supporting Information.

## Data Availability

Statistical maps from fMRI analyses will be added to Neurovault. All code used in this work to run analyses can be found in this open GitHub repository: https://github.com/daniellekurtin/MutualInformationFunctionalConnectivity_TaskSwitching.
