# Supplementary material for: Planning ahead: Predictable switching recruits task‐active and resting‐state networks
Source: Hum Brain Mapp. 2023 Jul 20;44(15):5030–46. doi: 10.1002/hbm.26430 (PMC10502652; doi:10.1002/hbm.26430)
Supplement: Supplementary file 1 — DATA S1 Supporting Information. [file HBM-44-5030-s001.docx]

# **Supplementary materials**


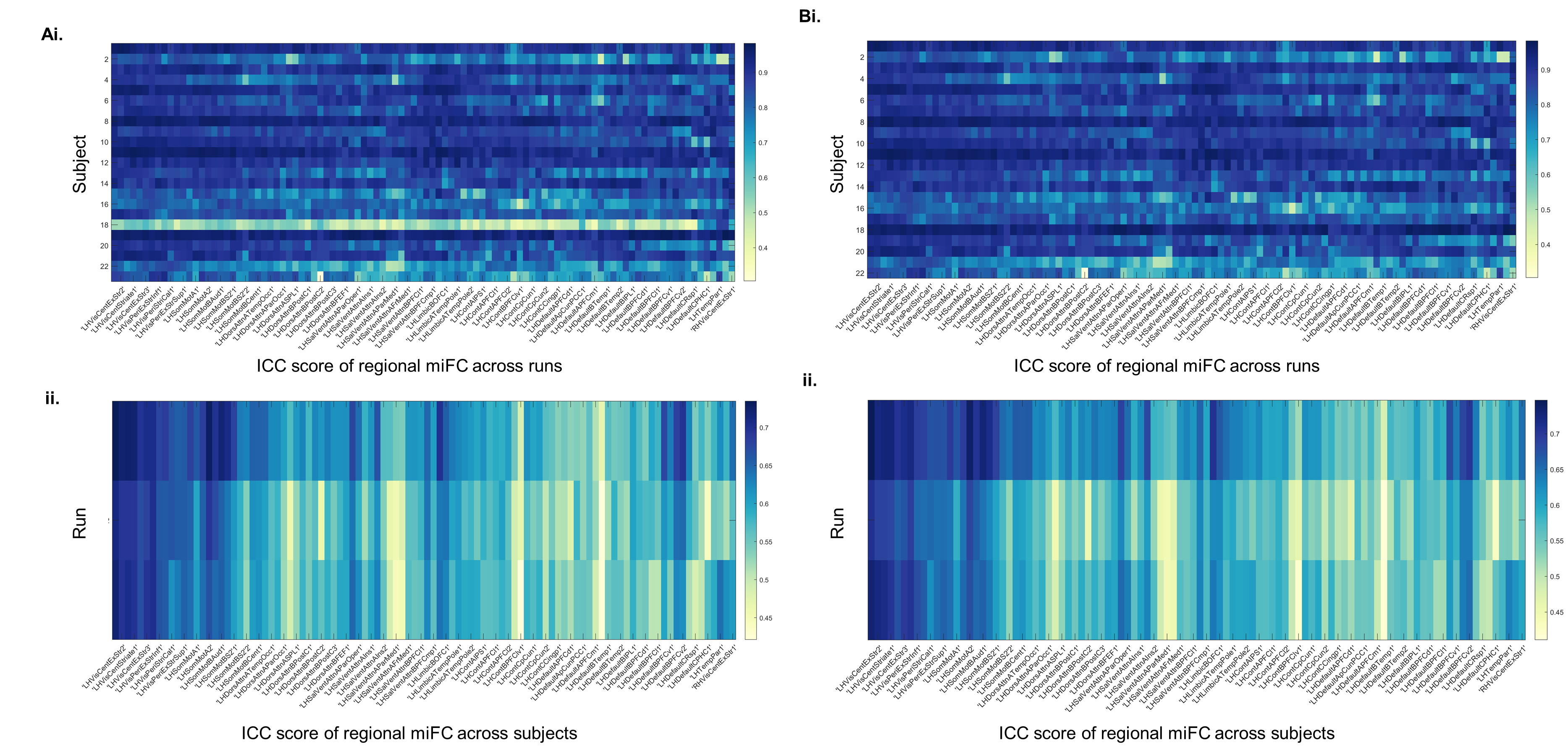


**Figure 1:** The ICC scores for regional miFC when run (A) with and (B) without subject 18. ICC was computed across (Ai) (Bi) runs and (Aii) (Bii) subjects.


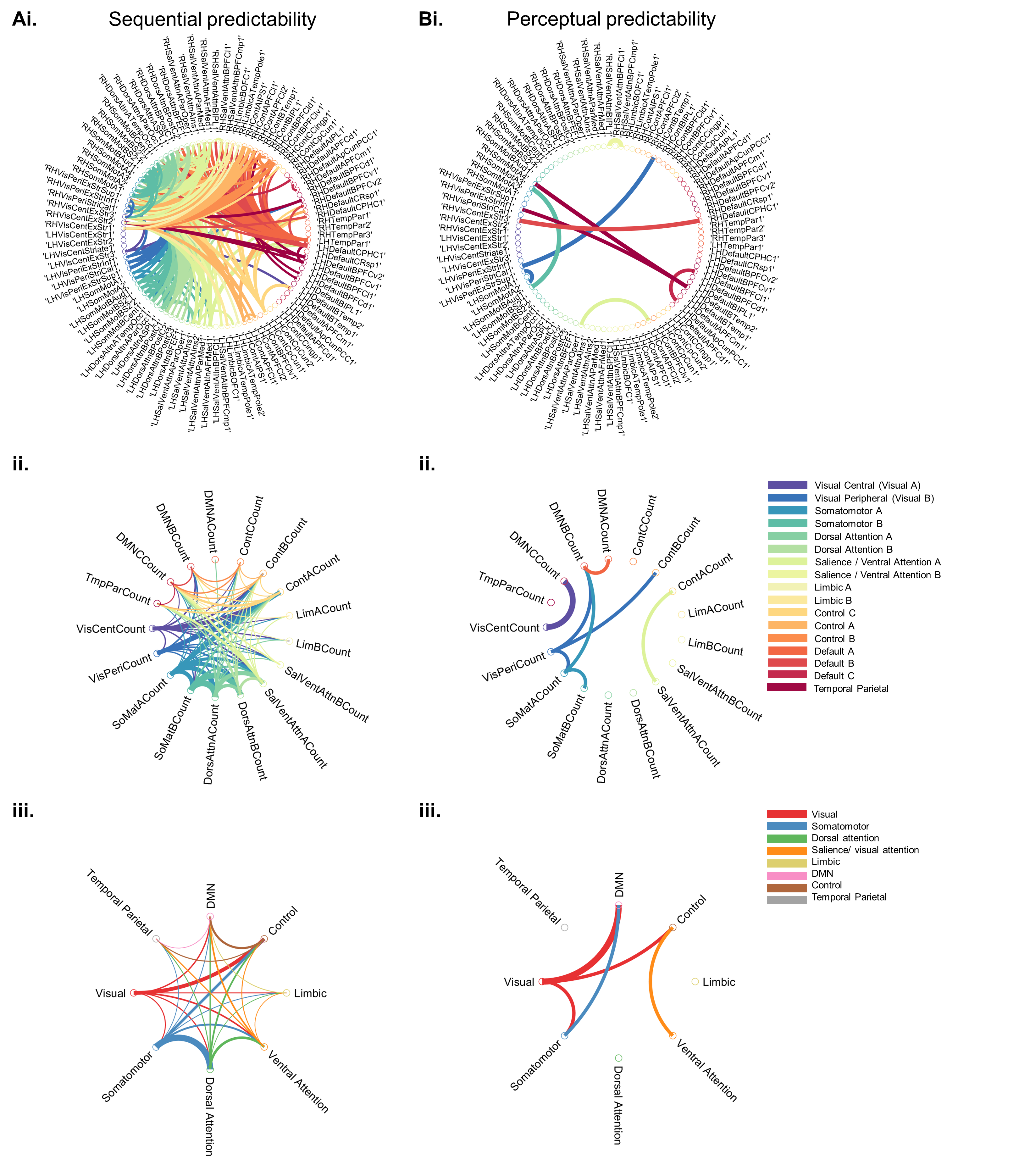


**Figure 2:** There were significant effects of **A** sequential **B** and perceptual predictability on miFC when the HRF was accounted for by shifting the timeseries 4 frames (8 seconds) as compared to the 5 frames (10 seconds) reported in the main text. Regions whose miFC was significantly influenced by **Ai.** sequential and **Bi.** perceptual predictability after FDR correction for multiple comparisons were connected between them, where the width of the connections was scaled to the effect size (η^2^). The first region out of the pair determines the colour of the connection, and colour is assigned according to the network the first region is in. Regions with significant changes in miFC due to **Aii.** sequential and **Bii.** perceptual predictability are grouped by network. The edges are scaled with their weight, which captures the proportion of connections between two connected network and the total number of edges. To aid visualization of networks with miFC influenced by **Aiii.** sequential **Biii.** perceptual predictability, we created graphs that grouped subnetworks together. For example, control networks A, B, and C were consolidated into one control network. Networks with a degree of 0 or within-network changes in miFC are not included in the network connectivity graphs.


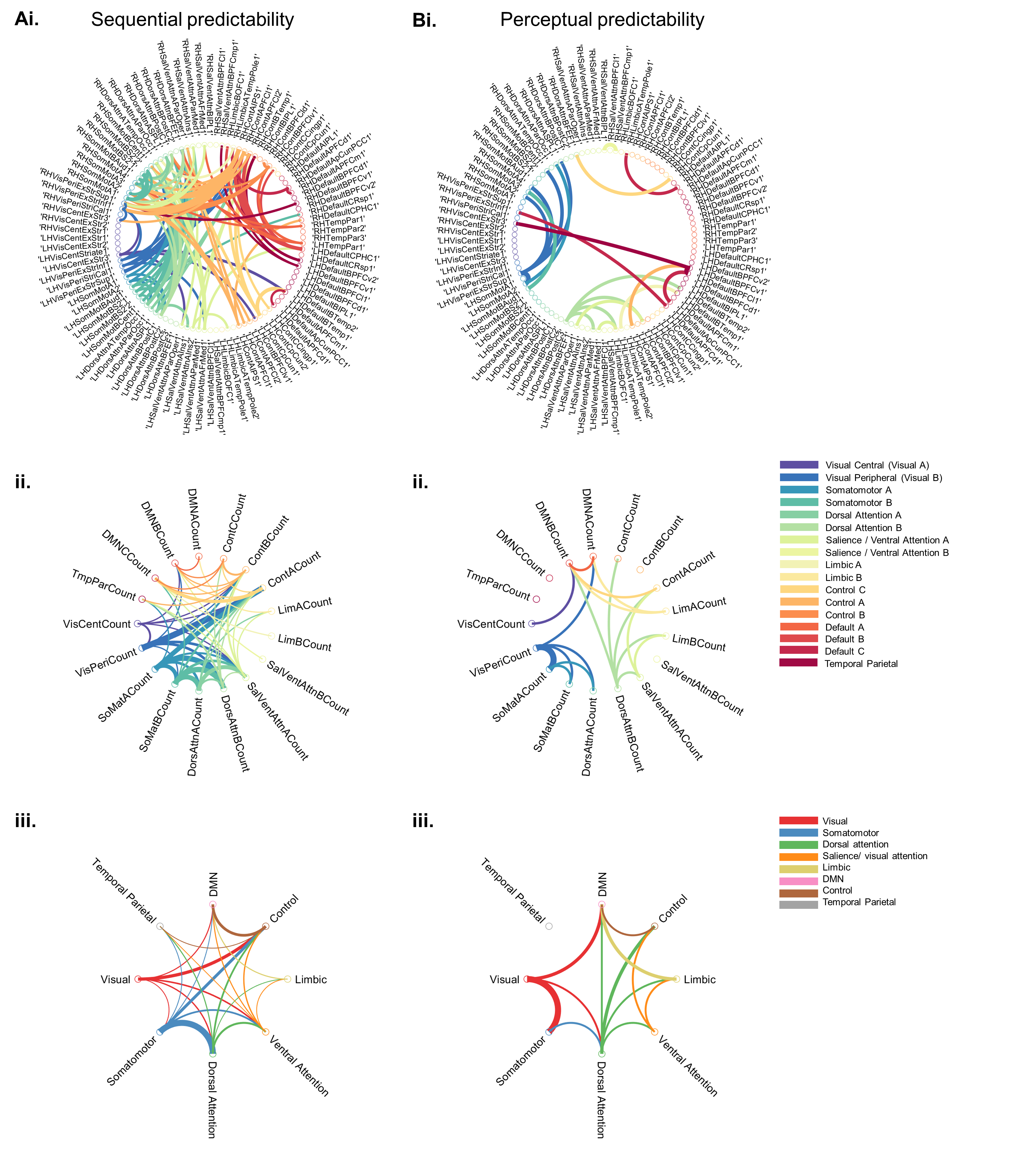


**Figure 3:** There were significant effects of **A** sequential **B** and perceptual predictability on miFC when the HRF was accounted for by shifting the timeseries 6 frames (12 seconds) as compared to the 5 frames (10 seconds) reported in the main text. Regions whose miFC was significantly influenced by **Ai.** sequential and **Bi.** perceptual predictability after FDR correction for multiple comparisons were connected between them, where the width of the connections was scaled to the effect size (η^2^). The first region out of the pair determines the colour of the connection, and colour is assigned according to the network the first region is in. Regions with significant changes in miFC due to **Aii.** sequential and **Bii.** perceptual predictability are grouped by network, where each node represents a network. The edges are scaled with their weight, which captures the proportion of connections between two connected network and the total number of edges. To aid visualization of networks with miFC influenced by **Aiii.** sequential **Biii.** perceptual predictability, we created graphs that grouped subnetworks together. For example, control networks A, B, and C were consolidated into one control network. Networks with a degree of 0 or within-network changes in miFC are not included in the network connectivity graphs.

**Table 1:** Results from repeated measures ANOVA showing a significant effect of sequential predictability on pairwise miFC. Post-hoc Wilcoxon signed rank tests show the direction of effects.

| **ROI** | **ROI** | **p** | **η^2^** | **Post hoc p** | **Post hoc z-stat** |
| --- | --- | --- | --- | --- | --- |
| RHSalVentAttnAParOper1 | LHSomMotBAud1 | 0.03 | 0.20 | 0.08 | 1.76 |
| RHSalVentAttnAParOper1 | LHSomMotBS2”1 | 0.05 | 0.17 | 0.07 | 1.79 |
| RHSalVentAttnAParOper1 | LHDorsAttnBPostC1 | 0.03 | 0.22 | 0.08 | 1.77 |
| RHSalVentAttnAParOper1 | LHSalVentAttnAParOper1 | 0.04 | 0.19 | 0.04 | 2.08 |
| RHSalVentAttnAIns1 | LHDorsAttnAParOcc1 | 0.04 | 0.18 | 0.17 | 1.37 |
| RHSalVentAttnAIns1 | LHDorsAttnASPL1 | 0.04 | 0.19 | 0.07 | 1.81 |
| RHSalVentAttnAParMed1 | LHDorsAttnBPostC1 | 0.03 | 0.22 | 0.08 | 1.77 |
| RHSalVentAttnAParMed1 | LHTempPar1 | 0.03 | 0.21 | 0.29 | 1.06 |
| RHSalVentAttnAParMed1 | RHVisCentExStr3 | 0.02 | 0.24 | 0.07 | 1.79 |
| RHSalVentAttnAParMed1 | RHSomMotA1 | 0.03 | 0.21 | 0.15 | 1.46 |
| RHSalVentAttnAParMed1 | RHSomMotA3 | 4.55E-02 | 0.18 | 0.21 | 1.25 |
| RHSalVentAttnAFrMed1 | LHTempPar1 | 0.02 | 0.24 | 0.26 | 1.12 |
| RHSalVentAttnAFrMed1 | RHVisPeriExStrSup1 | 0.04 | 0.18 | 0.15 | 1.44 |
| RHSalVentAttnAFrMed1 | RHSomMotA1 | 0.03 | 0.22 | 0.08 | 1.73 |
| RHSalVentAttnAFrMed1 | RHDorsAttnBPostC1 | 4.90E-02 | 0.17 | 0.05 | 1.95 |
| LHSalVentAttnAIns1 | LHDorsAttnASPL1 | 0.02 | 0.25 | 0.06 | 1.92 |
| LHSalVentAttnAFrMed1 | LHVisPeriStriCal1 | 0.02 | 0.23 | 0.08 | 1.72 |
| LHLimbicATempPole1 | LHSalVentAttnAIns2 | 0.03 | 0.20 | 0.14 | 1.46 |
| RHLimbicBOFC1 | RHVisPeriExStrInf1 | 0.04 | 0.18 | 0.30 | 1.03 |
| RHLimbicBOFC1 | RHSomMotA4 | 0.03 | 0.20 | 0.11 | 1.62 |
| LHTempPar1 | LHSalVentAttnAFrMed1 | 0.03 | 0.22 | 0.34 | 0.96 |
| LHDefaultAPFCd1 | LHLimbicBOFC1 | 0.04 | 0.18 | 0.23 | -1.21 |
| LHDefaultBTemp1 | LHVisCentExStr3 | 0.03 | 0.21 | 0.18 | -1.33 |
| LHDefaultBTemp1 | LHContCpCun1 | 0.01 | 0.29 | 0.06 | -1.85 |
| LHDefaultCPHC1 | LHSalVentAttnAFrMed1 | 0.01 | 0.30 | 0.11 | 1.58 |
| LHDefaultBPFCv2 | RHVisCentExStr2 | 4.61E-02 | 0.18 | 0.21 | 1.24 |
| LHDefaultBPFCv1 | RHSomMotA4 | 4.74E-02 | 0.17 | 0.23 | 1.20 |
| LHDefaultCPHC1 | RHSomMotBS2”1 | 0.03 | 0.20 | 0.14 | 1.48 |
| LHDefaultCPHC1 | RHDorsAttnBPostC1 | 0.01 | 0.31 | 0.19 | 1.31 |
| LHDefaultCPHC1 | RHDorsAttnASPL1 | 0.01 | 0.26 | 0.11 | 1.59 |
| LHDefaultCPHC1 | RHDorsAttnBPostC2 | 0.04 | 0.20 | 0.17 | 1.37 |
| LHDefaultCPHC1 | RHDorsAttnBFEF1 | 0.03 | 0.22 | 0.36 | 0.91 |
| LHDefaultCPHC1 | RHSalVentAttnAIns1 | 0.04 | 0.19 | 0.21 | 1.26 |
| LHDefaultCPHC1 | RHSalVentAttnBIPL1 | 0.04 | 0.19 | 0.38 | 0.89 |
| LHDefaultCPHC1 | RHSalVentAttnBPFCl1 | 0.02 | 0.25 | 0.14 | 1.49 |
| LHDefaultCPHC1 | RHSalVentAttnBPFCmp1 | 0.04 | 0.19 | 0.46 | 0.74 |
| LHDefaultCPHC1 | RHLimbicBOFC1 | 0.03 | 0.22 | 0.19 | 1.32 |
| LHDefaultCPHC1 | RHContAIPS1 | 2.37E-03 | 0.37 | 0.05 | 1.96 |
| LHDefaultCPHC1 | RHContAPFCl1 | 0.02 | 0.24 | 0.10 | 1.62 |
| LHDefaultCPHC1 | RHContAPFCl2 | 4.61E-03 | 0.33 | 0.05 | 2.00 |
| LHDefaultBPFCv2 | RHContBTemp1 | 0.03 | 0.21 | 4.95E-02 | 1.96 |
| LHDefaultCPHC1 | RHContBPFCld1 | 0.03 | 0.21 | 0.24 | 1.17 |
| LHDefaultCPHC1 | RHContBPFClv1 | 0.04 | 0.20 | 0.23 | 1.20 |
| LHDefaultBPFCv1 | RHSalVentAttnAParMed1 | 0.04 | 0.18 | 0.18 | 1.34 |
| RHDefaultApCunPCC1 | RHLimbicATempPole1 | 0.04 | 0.19 | 0.02 | 2.28 |
| RHDefaultAPFCm1 | RHDefaultApCunPCC1 | 0.04 | 0.19 | 0.05 | 1.99 |
| RHDefaultBPFCv1 | LHVisPeriExStrInf1 | 0.03 | 0.20 | 0.09 | 1.69 |
| RHDefaultBPFCv1 | RHVisPeriExStrSup1 | 0.03 | 0.21 | 0.06 | 1.85 |
| RHDefaultBPFCv2 | LHDorsAttnBFEF1 | 4.70E-02 | 0.17 | 0.06 | 1.89 |
| RHDefaultCPHC1 | LHSalVentAttnAIns1 | 4.56E-02 | 0.18 | 0.07 | 1.84 |
| RHDefaultCPHC1 | LHContAIPS1 | 0.02 | 0.23 | 0.07 | 1.83 |
| RHDefaultCPHC1 | LHContAPFCl2 | 5.00E-02 | 0.17 | 0.03 | 2.17 |
| RHDefaultCPHC1 | RHVisCentExStr2 | 0.04 | 0.18 | 0.03 | 2.18 |
| LHContAPFCl2 | LHContAIPS1 | 4.54E-02 | 0.18 | 0.07 | 1.81 |
| LHContBPFClv1 | LHVisCentExStr3 | 0.03 | 0.20 | 0.11 | -1.62 |
| RHContAIPS1 | LHVisPeriStriCal1 | 0.01 | 0.28 | 0.07 | 1.78 |
| RHContAIPS1 | LHSomMotA1 | 0.03 | 0.20 | 0.16 | 1.41 |
| RHContAIPS1 | LHDorsAttnBPostC1 | 0.04 | 0.19 | 0.30 | 1.03 |
| RHContAIPS1 | LHSalVentAttnAFrMed1 | 0.04 | 0.18 | 0.34 | 0.94 |
| RHContAIPS1 | LHContCpCun1 | 0.02 | 0.25 | 0.15 | 1.44 |
| RHContAIPS1 | LHTempPar1 | 4.58E-02 | 0.18 | 0.22 | 1.23 |
| RHContAIPS1 | RHVisCentExStr3 | 0.04 | 0.19 | 0.10 | 1.65 |
| RHContAIPS1 | RHVisPeriExStrInf1 | 0.01 | 0.32 | 0.09 | 1.68 |
| RHContAIPS1 | RHVisPeriExStrSup1 | 0.00 | 0.42 | 0.04 | 2.07 |
| RHContAIPS1 | RHSomMotA1 | 0.04 | 0.19 | 0.24 | 1.18 |
| RHContAIPS1 | RHSomMotA4 | 4.91E-03 | 0.33 | 0.07 | 1.79 |
| RHContAIPS1 | RHSomMotBS2”1 | 0.02 | 0.23 | 0.16 | 1.40 |
| RHContAIPS1 | RHSomMotBCent1 | 0.03 | 0.20 | 0.32 | 1.00 |
| RHContAPFCl1 | LHVisPeriStriCal1 | 4.77E-02 | 0.17 | 0.14 | 1.47 |
| RHContAPFCl1 | RHVisCentExStr3 | 0.03 | 0.20 | 0.09 | 1.71 |
| RHContAPFCl1 | RHVisPeriExStrInf1 | 0.01 | 0.30 | 0.08 | 1.74 |
| RHContAPFCl1 | RHVisPeriExStrSup1 | 0.01 | 0.32 | 0.07 | 1.83 |
| RHContAPFCl1 | RHSomMotA2 | 0.04 | 0.20 | 0.10 | 1.63 |
| RHContAPFCl1 | RHSomMotA4 | 0.04 | 0.19 | 0.16 | 1.39 |
| RHContAPFCl2 | LHVisPeriExStrInf1 | 0.03 | 0.20 | 0.21 | 1.24 |
| RHContAPFCl2 | LHDorsAttnAParOcc1 | 0.03 | 0.20 | 0.10 | 1.65 |
| RHContAPFCl2 | LHDorsAttnASPL1 | 0.04 | 0.19 | 0.12 | 1.54 |
| RHContAPFCl2 | LHDorsAttnBPostC1 | 0.03 | 0.20 | 0.18 | 1.35 |
| RHContAPFCl2 | LHTempPar1 | 0.04 | 0.19 | 0.21 | 1.26 |
| RHContAPFCl2 | RHVisCentExStr1 | 0.04 | 0.19 | 0.14 | 1.49 |
| RHContAPFCl2 | RHVisCentExStr3 | 0.03 | 0.20 | 0.03 | 2.22 |
| RHContAPFCl2 | RHVisPeriExStrInf1 | 0.04 | 0.19 | 0.29 | 1.07 |
| RHContAPFCl2 | RHSomMotA4 | 0.03 | 0.21 | 0.20 | 1.28 |
| RHContAPFCl2 | RHContAPFCl1 | 0.04 | 0.18 | 0.14 | 1.47 |
| RHContBTemp1 | LHSalVentAttnAIns2 | 0.04 | 0.19 | 0.16 | 1.40 |
| RHContBTemp1 | LHContCpCun1 | 0.02 | 0.22 | 0.06 | 1.87 |
| RHContBPFClv1 | LHTempPar1 | 0.03 | 0.20 | 0.18 | 1.35 |
| RHContBPFClv1 | RHVisPeriExStrInf1 | 0.02 | 0.23 | 0.24 | 1.17 |
| RHContBPFClv1 | RHVisPeriExStrSup1 | 0.01 | 0.27 | 0.14 | 1.46 |
| RHContBPFClv1 | RHSomMotA1 | 0.01 | 0.26 | 0.14 | 1.49 |
| RHContBPFClv1 | RHSomMotA2 | 0.01 | 0.27 | 0.10 | 1.62 |
| RHContBPFClv1 | RHDorsAttnBPostC1 | 4.54E-02 | 0.18 | 0.18 | 1.35 |
| RHContBPFClv1 | RHContBPFCld1 | 0.02 | 0.25 | 0.05 | 1.95 |
| RHVisCentExStr2 | LHSalVentAttnAFrMed1 | 0.04 | 0.19 | 0.26 | 1.13 |
| RHVisCentExStr3 | LHSalVentAttnAFrMed1 | 4.83E-02 | 0.17 | 0.10 | 1.66 |
| RHVisPeriExStrInf1 | LHVisCentExStr3 | 0.04 | 0.18 | 0.08 | 1.72 |
| RHVisPeriExStrInf1 | LHVisPeriExStrInf1 | 0.02 | 0.23 | 4.67E-02 | 1.99 |
| RHVisPeriExStrInf1 | RHVisCentExStr3 | 0.02 | 0.22 | 0.10 | 1.64 |
| RHVisPeriExStrSup1 | RHVisPeriExStrSup1 | 0.04 | 0.19 | 0.04 | 2.06 |
| LHVisCentExStr3 | LHVisCentExStr3 | 0.01 | 0.26 | 0.02 | 2.27 |
| LHVisPeriExStrInf1 | LHVisPeriExStrInf1 | 0.02 | 0.24 | 0.06 | 1.92 |
| LHVisPeriStriCal1 | LHVisPeriStriCal1 | 0.02 | 0.25 | 0.03 | 2.16 |
| RHSomMotA1 | LHSalVentAttnAFrMed1 | 0.04 | 0.19 | 0.19 | 1.32 |
| RHSomMotA1 | LHContAPFCl2 | 0.02 | 0.22 | 0.19 | 1.31 |
| RHSomMotA1 | LHContCpCun1 | 0.04 | 0.19 | 0.13 | 1.52 |
| RHSomMotA2 | LHSomMotBCent1 | 0.04 | 0.19 | 0.11 | 1.61 |
| RHSomMotA2 | LHDorsAttnBPostC1 | 0.02 | 0.22 | 0.07 | 1.78 |
| RHSomMotA2 | LHContAIPS1 | 0.04 | 0.19 | 0.08 | 1.75 |
| RHSomMotA2 | LHContCpCun1 | 0.04 | 0.18 | 0.08 | 1.77 |
| RHSomMotA4 | LHDorsAttnBPostC1 | 0.02 | 0.23 | 0.12 | 1.54 |
| RHSomMotA4 | LHSalVentAttnAFrMed1 | 0.03 | 0.20 | 0.12 | 1.54 |
| RHSomMotA4 | LHContAIPS1 | 0.04 | 0.19 | 0.15 | 1.43 |
| RHSomMotA4 | LHTempPar1 | 0.03 | 0.20 | 0.15 | 1.42 |
| RHSomMotA4 | RHVisCentExStr3 | 0.04 | 0.20 | 0.40 | 0.84 |
| RHSomMotBAud1 | RHSomMotA1 | 0.01 | 0.27 | 0.04 | 2.05 |
| RHSomMotBS2”1 | LHSomMotBCent1 | 0.03 | 0.20 | 0.12 | 1.54 |
| RHSomMotBS2”1 | LHDorsAttnASPL1 | 0.04 | 0.18 | 0.13 | 1.53 |
| RHSomMotBS2”1 | LHDorsAttnBPostC1 | 0.03 | 0.22 | 0.08 | 1.78 |
| RHSomMotBS2”1 | RHVisCentExStr3 | 0.04 | 0.18 | 0.19 | 1.31 |
| RHSomMotBS2”1 | RHSomMotA1 | 3.43E-03 | 0.35 | 0.01 | 2.77 |
| RHSomMotBS2”1 | RHSomMotA2 | 0.01 | 0.26 | 0.13 | 1.52 |
| RHSomMotBS2”1 | RHSomMotA4 | 0.02 | 0.25 | 0.07 | 1.80 |
| RHSomMotBS2”2 | RHSomMotA1 | 0.03 | 0.22 | 0.04 | 2.03 |
| RHSomMotBS2”2 | RHSomMotA4 | 0.04 | 0.18 | 0.11 | 1.59 |
| RHSomMotBS2”2 | RHSomMotBS2”1 | 0.03 | 0.20 | 0.03 | 2.13 |
| RHSomMotBCent1 | LHVisPeriStriCal1 | 0.03 | 0.20 | 0.11 | 1.58 |
| RHSomMotBCent1 | LHSomMotBS2”2 | 0.03 | 0.20 | 4.72E-02 | 1.98 |
| RHSomMotBCent1 | LHSomMotBCent1 | 0.03 | 0.21 | 0.06 | 1.91 |
| RHSomMotBCent1 | LHDorsAttnATempOcc1 | 0.02 | 0.25 | 0.06 | 1.89 |
| RHSomMotBCent1 | LHDorsAttnBPostC1 | 0.01 | 0.28 | 0.02 | 2.39 |
| RHSomMotBCent1 | LHSalVentAttnAFrMed1 | 4.62E-02 | 0.18 | 0.08 | 1.74 |
| RHSomMotBCent1 | RHSomMotBS2”1 | 0.04 | 0.19 | 0.10 | 1.64 |
| RHDorsAttnATempOcc1 | LHSomMotA2 | 0.04 | 0.18 | 0.08 | 1.75 |
| RHDorsAttnATempOcc1 | LHSomMotBS2”2 | 0.03 | 0.20 | 0.11 | 1.59 |
| RHDorsAttnATempOcc1 | LHDorsAttnATempOcc1 | 0.01 | 0.32 | 0.01 | 2.62 |
| RHDorsAttnATempOcc1 | LHDorsAttnAParOcc1 | 0.04 | 0.18 | 0.04 | 2.09 |
| RHDorsAttnATempOcc1 | LHDorsAttnBPostC1 | 4.95E-02 | 0.17 | 0.05 | 1.93 |
| RHDorsAttnATempOcc1 | LHDorsAttnBFEF1 | 0.04 | 0.18 | 0.04 | 2.04 |
| RHDorsAttnATempOcc1 | LHSalVentAttnAFrMed1 | 0.02 | 0.23 | 4.86E-02 | 1.97 |
| RHDorsAttnATempOcc1 | LHLimbicATempPole1 | 4.81E-02 | 0.17 | 0.15 | 1.43 |
| RHDorsAttnAParOcc1 | RHSomMotA4 | 0.03 | 0.22 | 0.11 | 1.62 |
| RHDorsAttnASPL1 | LHSomMotA1 | 0.03 | 0.21 | 0.09 | 1.71 |
| RHDorsAttnASPL1 | LHSomMotA2 | 0.04 | 0.18 | 0.08 | 1.76 |
| RHDorsAttnASPL1 | LHSomMotBAud1 | 0.02 | 0.23 | 0.19 | 1.31 |
| RHDorsAttnASPL1 | LHSomMotBS2”2 | 4.08E-02 | 0.19 | 1.34E-01 | 1.50 |
| RHDorsAttnASPL1 | LHSomMotBCent1 | 0.03 | 0.22 | 0.08 | 1.78 |
| RHDorsAttnASPL1 | LHDorsAttnATempOcc1 | 0.01 | 0.27 | 0.08 | 1.73 |
| RHDorsAttnASPL1 | LHTempPar1 | 0.03 | 0.21 | 0.13 | 1.52 |
| RHDorsAttnASPL1 | RHVisPeriExStrInf1 | 0.02 | 0.23 | 0.12 | 1.56 |
| RHDorsAttnASPL1 | RHSomMotA2 | 0.04 | 0.18 | 0.15 | 1.45 |
| RHDorsAttnASPL1 | RHSomMotA4 | 4.61E-03 | 0.33 | 0.07 | 1.83 |
| RHDorsAttnASPL1 | RHSomMotBS2”1 | 0.02 | 0.25 | 0.13 | 1.53 |
| RHDorsAttnASPL1 | RHSomMotBCent1 | 0.04 | 0.19 | 0.19 | 1.30 |
| RHDorsAttnBPostC1 | LHSomMotBCent1 | 0.02 | 0.24 | 0.04 | 2.09 |
| RHDorsAttnBPostC1 | RHSomMotBS2”1 | 0.01 | 0.27 | 0.03 | 2.19 |
| RHDorsAttnBPostC2 | LHSomMotBAud1 | 4.95E-02 | 0.17 | 0.11 | 1.58 |
| RHDorsAttnBFEF1 | LHVisPeriExStrInf1 | 0.03 | 0.21 | 0.15 | 1.45 |
| RHDorsAttnBFEF1 | LHVisPeriStriCal1 | 0.02 | 0.24 | 0.10 | 1.66 |
| RHDorsAttnBFEF1 | LHVisPeriExStrSup1 | 0.00 | 0.35 | 0.01 | 2.61 |
| RHDorsAttnBFEF1 | LHSomMotA1 | 0.03 | 0.21 | 0.16 | 1.40 |
| RHDorsAttnBFEF1 | LHSomMotBAud1 | 0.01 | 0.26 | 0.03 | 2.14 |
| RHDorsAttnBFEF1 | LHSomMotBS2”1 | 4.89E-02 | 0.17 | 0.12 | 1.55 |
| RHDorsAttnBFEF1 | LHSomMotBCent1 | 0.01 | 0.27 | 0.06 | 1.90 |
| RHDorsAttnBFEF1 | LHDorsAttnATempOcc1 | 0.03 | 0.20 | 0.08 | 1.78 |
| RHDorsAttnBFEF1 | LHDorsAttnASPL1 | 0.01 | 0.26 | 0.11 | 1.61 |
| RHDorsAttnBFEF1 | LHContAIPS1 | 0.04 | 0.18 | 0.36 | 0.92 |
| RHDorsAttnBFEF1 | LHContCpCun1 | 0.02 | 0.23 | 0.23 | 1.21 |
| RHDorsAttnBFEF1 | LHTempPar1 | 0.02 | 0.25 | 0.18 | 1.33 |
| RHDorsAttnBFEF1 | RHSomMotA1 | 0.02 | 0.22 | 0.09 | 1.71 |
| RHDorsAttnBFEF1 | RHSomMotA4 | 0.01 | 0.28 | 0.09 | 1.71 |
| LHDorsAttnATempOcc1 | LHSomMotA2 | 0.02 | 0.23 | 0.06 | 1.88 |
| LHDorsAttnATempOcc1 | LHSomMotBS2”2 | 0.03 | 0.21 | 0.03 | 2.22 |
| LHDorsAttnATempOcc1 | LHDorsAttnATempOcc1 | 0.04 | 0.19 | 0.12 | 1.54 |
| LHDorsAttnASPL1 | LHSomMotA2 | 0.04 | 0.19 | 0.07 | 1.83 |
| LHDorsAttnASPL1 | LHSomMotBAud1 | 0.01 | 0.26 | 0.10 | 1.66 |
| LHDorsAttnASPL1 | LHSomMotBS2”1 | 4.70E-02 | 0.18 | 0.12 | 1.56 |
| LHDorsAttnBPostC1 | LHSomMotA1 | 0.02 | 0.23 | 0.07 | 1.79 |
| LHDorsAttnBPostC1 | LHSomMotA2 | 0.01 | 0.26 | 0.04 | 2.03 |
| LHDorsAttnBPostC1 | LHSomMotBS2”1 | 0.01 | 0.30 | 0.04 | 2.10 |
| LHDorsAttnBPostC1 | LHSomMotBS2”2 | 0.04 | 0.18 | 0.10 | 1.66 |
| LHDorsAttnBPostC1 | LHDorsAttnASPL1 | 0.03 | 0.21 | 0.05 | 1.93 |
| LHDorsAttnBPostC2 | LHSomMotA2 | 0.04 | 0.20 | 0.11 | 1.58 |

**Table 2:** Results from repeated measures ANOVA showing a significant effect of perceptual predictability on pairwise miFC. Post-hoc Wilcoxon signed rank tests show the direction of effects.

| **ROI** | **ROI** | **p** | **η^2^** | **Post hoc p** | **Post hoc z-stat** |
| --- | --- | --- | --- | --- | --- |
| LHSomMotA2 | LHVisPeriExStrSup1 | 0.03 | 0.20 | 4.54E-02 | -2.00 |
| RHSomMotA3 | LHSomMotBAud1 | 0.04 | 0.19 | 0.07 | -1.78 |
| RHSomMotA4 | LHVisPeriExStrSup1 | 0.03 | 0.20 | 0.06 | -1.88 |
| RHSomMotBCent1 | LHVisPeriExStrSup1 | 0.04 | 0.18 | 0.07 | -1.84 |
| RHSalVentAttnBPFCmp1 | RHSalVentAttnBIPL1 | 0.02 | 0.25 | 0.01 | 2.49 |
| RHContAIPS1 | LHVisCentExStr1 | 0.04 | 0.18 | 0.07 | -1.84 |
| RHContCpCun1 | RHDorsAttnBFEF1 | 0.04 | 0.19 | 0.01 | 2.47 |
| RHDefaultCPHC1 | RHDefaultCPHC1 | 0.04 | 0.18 | 0.07 | 1.83 |
| LHDefaultBPFCl1 | LHDefaultApCunPCC1 | 0.03 | 0.21 | 0.06 | -1.92 |
| LHDefaultBTemp2 | RHVisPeriExStrInf1 | 4.79E-02 | 0.17 | 0.20 | -1.29 |

**Table 3:** Graph theoretic metrics for changes in miFC within 17 functional networks due to sequential predictability.

| **Network name** | **Degree** | **Proportion of total edges** |
| --- | --- | --- |
| Somatomotor B | 46 | 0.13 |
| Somatomotor A | 45 | 0.12 |
| Dorsal Attention A | 42 | 0.12 |
| Control A | 41 | 0.11 |
| Dorsal Attention B | 39 | 0.11 |
| Salience/Ventral Attention A | 32 | 0.09 |
| Visual Peripheral | 30 | 0.08 |
| DMN C | 20 | 0.05 |
| Visual Central | 17 | 0.05 |
| Control B | 14 | 0.04 |
| DMN B | 9 | 0.02 |
| Temporal Parietal | 9 | 0.02 |
| Control C | 6 | 0.02 |
| Limbic B | 4 | 0.01 |
| DMN A | 4 | 0.01 |
| Salience/Ventral Attention B | 3 | 0.01 |
| Limbic A | 3 | 0.01 |

**Table 4:** Graph theoretic metrics for changes in miFC within the reduced set of 8 functional networks due to sequential predictability.

| **Network name** | **Degree** | **Proportion of total edges** |
| --- | --- | --- |
| Somatomotor | 91 | 0.25 |
| Dorsal Attention | 81 | 0.22 |
| Control | 61 | 0.17 |
| Visual | 47 | 0.13 |
| Salience/ Ventral Attention | 35 | 0.10 |
| DMN | 33 | 0.09 |
| Temporal Parietal | 9 | 0.02 |
| Limbic | 7 | 0.02 |

**Table 5:** Graph theoretic metrics for changes in miFC within 17 functional networks due to perceptual predictability. Networks with a degree of 0 are not included in the table.

| **Network name** | **Degree** | **Proportion of total edges** |
| --- | --- | --- |
| Visual Peripheral | 4 | 0.20 |
| Somatomotor A | 3 | 0.15 |
| Somatomotor B | 2 | 0.10 |
| Salience/Ventral Attention B | 2 | 0.10 |
| DMN B | 2 | 0.10 |
| DMN C | 2 | 0.10 |
| Visual Central | 1 | 0.05 |
| Dorsal Attention B | 1 | 0.05 |
| Control A | 1 | 0.05 |
| Control C | 1 | 0.05 |
| DMN A | 1 | 0.05 |

**Table 6:** Graph theoretic metrics for changes in miFC within the reduced set of 8 functional networks due to perceptual predictability. Networks with a degree of 0 are not included in the table.

| **Network name** | **Degree** | **Proportion of total edges** |
| --- | --- | --- |
| Visual | 5 | 0.25 |
| Somatomotor | 5 | 0.25 |
| DMN | 5 | 0.25 |
| Salience/Ventral Attention | 2 | 0.10 |
| Control | 2 | 0.10 |
| Dorsal Attention | 1 | 0.05 |
